# Bioactive profiles of edible vegetable oils determined using 10D hyphenated comprehensive high-performance thin-layer chromatography (HPTLC×HPTLC) with on-surface metabolism (nanoGIT) and planar bioassays

**DOI:** 10.3389/fnut.2023.1227546

**Published:** 2023-09-22

**Authors:** Isabel Müller, Alexander Gulde, Gertrud E. Morlock

**Affiliations:** ^1^Institute of Nutritional Science, Chair of Food Science, as well as Interdisciplinary Research Centre for Biosystems, Land Use and Nutrition, Justus Liebig University Giessen, Giessen, Germany; ^2^Center for Sustainable Food Systems, Justus Liebig University Giessen, Giessen, Germany

**Keywords:** all-in-one digestion analysis system, on-surface metabolization, lipolysis, effect-directed analysis, intestinal phase, comprehensive high-performance thin-layer chromatography, plant oils, sustainability transition

## Abstract

**Introduction:**

Vegetable oils rich in unsaturated fatty acids are assumed to be safe and even healthy for consumers though lipid compositions of foods vary naturally and are complex considering the wealth of minor compounds down to the trace level.

**Methods:**

The developed comprehensive high-performance thin-layer chromatography (HPTLC×HPTLC) method including the on-surface metabolization (nanoGIT) and bioassay detection combined all steps on the same planar surface. The pancreatic lipolysis (intestinal phase) experiment and the subsequent analysis of the fatty acid composition including its effect-directed detection using a planar bioassay was performed without elaborate sample preparation or fractionation to ensure sample integrity. Thus, no sample part was lost, and the whole sample was studied on a single surface regarding all aspects. This made the methodology as well as technology miniaturized, lean, all-in-one, and very sustainable.

**Results and discussion:**

To prioritize important active compounds including their metabolism products in the complex oil samples, the nanoGIT method was used to examine the pancreatic lipolysis of nine different vegetable oils commonly used in the kitchen and food industry, e.g., canola oil, flaxseed oil, hemp oil, walnut oil, soybean oil, sunflower oil, olive oil, coconut oil, and palm oil. The digested oils revealed antibacterial and genotoxic effects, which were assigned to fatty acids and oxidized species via high-resolution tandem mass spectrometry (HRMS/MS). This finding reinforces the importance of adding powerful techniques to current analytical tools. The 10D hyphenated nanoGIT-HPTLC×HPTLC-Vis/FLD-bioassay-heart cut-RP-HPLC-DAD-HESI-HRMS/MS has the potential to detect any potential hazard due to digestion/metabolism, improving food safety and understanding on the impact of complex samples.

## 1. Introduction

Lipids are one of the three important macronutrients in the human diet, and fat has the highest energy density of all nutrients. It is recommended that not more than 30%−35% of the energy intake should be in the form of fat (1, 2). The fatty acid (FA) composition of dietary fats can influence body weight (3, 4). High intake of saturated or monounsaturated fats causes an increase in weight gain and waist circumference, a factor for adiposity, whereas polyunsaturated fatty acids (PUFAs) show no increase. Among other aspects, saturated fatty acids (SFAs) such as lauric acid (C12:0), myristic acid (C14:0), and palmitic acid (C16:0) are associated with a higher risk of cardiovascular disease (5, 6). Replacing SFA-rich fats with PUFA-rich oils showed a lower risk of cardiovascular disease but no effect on adiposity (7), as mentioned earlier. Therefore, a deeper knowledge of the FA composition of food and its impact is of the utmost interest since the biological activity of FAs may influence not only cell and tissue metabolism and signaling pathways (8) but also our microbiome (9) and health (10). To achieve a low-fat diet, foods of plant origin are preferred to those of animal origin (1). Such plant-based foods contain less total fat and a more favorable FA composition, such as more essential FAs, namely α-linolenic acid (C18:3, *n*-3) and linoleic acid (C18:2, *n*-6), whereby *n*-6 to *n*-3 FA ratios of 1–5:1 are preferred (4, 11, 12); however, the ratio alone is not decisive for a diet recommendation (13, 14). Therefore, edible vegetable oils are the most commonly used fats in the kitchen and for the preparation and processing of foods (15, 16). They mainly consist of triacylglycerols (TAGs), in addition to a few percent of diacylglycerols (DAGs), monoacylglycerols (MAGs), and free FAs, as well as further lipophilic minor compounds in the per mille range down to the trace level (17–19).

The simulation of *in vitro* digestion is important to provide insights into the digestion mechanisms of fats and oils. After partial hydrolysis of TAGs by gastric lipases, the nutrients entering the small intestine are emulsified by bile salts and further digested enzymatically by pancreatic lipases in the intestinal phase (20). Both lipases cleave TAGs mainly into 2-MAGs and free FAs. However, while the main hydrolysis takes place in the intestine, it should not be neglected that gastric and pancreatic lipases act as complementary enzymes (21). The rate of lipolysis of TAGs is dependent on the FA chain length and degree of saturation (22–24). Research is currently being conducted on various simulated digestion models to study the digestibility of isolated nutrients in foods or the food itself, not ignoring the influence of the food matrix (25, 26). Due to the sensitivity of simulated digestion systems to altering enzymatic parameters and environmental conditions, Minekus et al. (27) designed an internationally harmonized protocol for static *in vitro* digestion via oral, gastric, and intestinal phases. Morlock et al. (28) showed the successful transfer of the internationally harmonized protocol for *in vitro* assays to high-performance thin-layer chromatography (HPTLC–UV/Vis/FLD), and moreover, they created an all-in-one digestion and analysis system for on-surface digestion at the nanomolar level (nanoGIT), followed by the analysis of the food samples, including the resulting metabolism products, all on the same surface. The optional hyphenation with post-chromatographic derivatization reagents, planar bioassays, and high-resolution tandem mass spectrometry (HRMS/MS) makes the lean all-in-one methodology very flexible, fast, and sustainable. In contrast, all current methods require elaborate sample preparation after the simulated lipolysis and the subsequent analysis of the metabolized food samples. Mostly, spectrophotometric assay kits or titration methods, such as the pH-stat method, are used for the determination of the sum of hydrolyzed FAs and thus lipolysis rate (26, 29, 30). Gas chromatography and high-performance liquid chromatography methods are performed rarely (23, 31), although Helbig et al. (29) showed the necessity of examining the detailed FA composition.

In this study, an all-in-one 10D hyphenated nanoGIT–HPTLC×HPTLC–Vis/FLD–bioassay–heart cut–RP-HPLC–DAD–HESI-HRMS/MS methodology was created and studied for the first time. The nanoGIT system was used to examine the pancreatic lipolysis of nine different vegetable oils commonly used in the kitchen and food industry, i.e., canola oil, flaxseed oil, hemp oil, walnut oil, soybean oil, sunflower oil, olive oil, coconut oil, and palm oil. A two-dimensional (2D) separation with orthogonal selectivity was developed for the differentiation of the lipids, resulting in a comprehensive HPTLC×HPTLC method. The entire sample separated in the first dimension was transferred to the second orthogonal separation dimension by a simple 90° plate turn. After the on-surface nanoGIT digestion, the first dimension was separated based on functional groups such as TAGs, DAGs, MAGs, and FAs. In the second separation dimension, the FAs were separated according to lipophilicity, and the approximate FA composition was determined. Antibacterial and genotoxic properties of the lipids were detected via respective bioassays and assigned to molecules via automated heart cuts of the active zones of interest to RP-HPLC–DAD–HESI-HRMS/MS. No information or sample part was lost since the whole workflow was performed on the same planar surface.

## 2. Materials and methods

### 2.1. Chemicals and materials

3-(4,5-Dimethylthiazol-2-yl)-2,5-diphenyltetrazolium bromide (MTT, ≥98%), acetone (≥99.9%), formic acid (≥99.9%), acetic acid (100%), dipotassium phosphate trihydrate (≥99.9%), glycerol (86%), monopotassium phosphate (≥99%), magnesium chloride (≥98.5%), sodium chloride (≥99.8%), monosodium phosphate monohydrate (≥98%), *n*-hexane (≥98%), sulfuric acid (96%, *p*. *a*.), decanoic acid (C10:0, >98%), octanoic acid (C8:0, >99%), oleic acid (C18:1, >99%), stearic acid (C18:0, >98%), sodium hydroxide (≥98%), 4-methylumbelliferyl-β-d-galactopyranoside (≥99%, for biochemistry), dimethylsulfoxide (≥99.8%), and molecular sieve 3 Å (0.3 nm, type 564, beads) were purchased from Carl Roth (Karlsruhe, Germany). Acetonitrile (≥99.9%), disodium phosphate (≥99%), sodium bicarbonate (≥99.7%), pancreatin from porcine pancreas (8 × USP specifications), bile extract porcine, dioleoylglycerol (diolein, >99%, mixture of 1,3- and 1,2-isomers), glyceryl trioleate (triolein, ≥99%), 1-oleoyl-*rac*-glycerol (monoolein, >99%; 2-monoolein was rarely available and six times more expensive), caffeine (>99%), linoleic acid (C18:2, 60%−74%), myristic acid (C14:0, >99%), palmitic acid (C16:0, >99%), dodecanoic acid (C12:0, 98%), peptone from casein (for microbiology), Müller–Hinton broth (for microbiology), d-(+)-glucose (99.5%), ampicillin sodium salt, and lysogeny broth (LB) powder (including 5 g/L of sodium chloride) were purchased from Fluka-Sigma-Aldrich (Steinheim, Germany). Methanol (99.9%) was supplied by VWR International (Darmstadt, Germany). Magnesium sulfate heptahydrate (99.5%), potassium chloride (≥99.5%), citric acid monohydrate (≥99.5%), HPTLC plates silica gel 60 RP-18 W, HPTLC plates silica gel 60 RP-18, and HPTLC plates silica gel 60 as cover plates (all 20 cm × 10 cm), and *Bacillus subtilis* subsp. *spizizenii* spore suspension (DSM 618) were purchased from Merck (Darmstadt, Germany). Diammonium phosphate (≥99%), diethyl ether (≥99%), and linolenic acid (C18:3, 99%) were purchased from Acros Organics (Morris Plains, NJ, USA). Yeast extract powder (for microbiology), ethyl acetate (≥99.8%), *o*-phosphoric acid (85%), ethanol (≥99.9%), and dichloromethane (≥99.9%, stabilized with amylene) were purchased from Th. Geyer (Renningen, Germany). Copper(II) sulfate pentahydrate (p. a.) was purchased from Honeywell International (Morristown, NJ, USA). Calcium chloride dihydrate (≥99.9%) was supplied by Bernd Kraft (Duisburg, Germany). The luminescent marine *Aliivibrio fischeri* (DSM 7151) bacteria were purchased from the DSMZ Leibnitz Institut (Berlin, Germany). Tetracycline hydrochloride (research grade, USP) was purchased from Serva Electrophoresis (Heidelberg, Germany). Bidistilled water was produced by a Heraeus Destamat B-18E (Thermo Fisher Scientific, Dreieich, Germany). Rhodamine 6G (100% ± 3%) was purchased from Alfa Aesar (Kandel, Germany). *Salmonella typhimurium* bacteria strain TA1535, modified to contain the plasmid pSK1002 (PTM *S. typhimurium* TA1535/pSK1002, cryostock), was purchased from Trinova Biochem (Giessen, Germany). 4-Nitroquinoline-1-oxide (98%) was purchased from TCI (Eschborn, Germany). Edible vegetable oils (Supplementary Table S1) were purchased from local supermarkets.

### 2.2. Pre-treatment of the HPTLC RP-18 W plate

For on-surface metabolic reactions, the HPTLC RP-18 W plate was pre-treated as follows. The plate was heated at 120°C for 60 min (TLC Plate Heater III, CAMAG, Muttenz, Switzerland; to fix the binder for the current plate batches used) and pre-washed by development first with methanol and then, after plate drying, with ethyl acetate, both up to 90 mm in a twin-trough chamber. To ensure the pancreatin reaction in the application zone, the acidic plate pH (ca. pH 4.2) was neutralized via piezoelectrical spraying (2.8 ml, ultra-yellow nozzle, level 3, Derivatizer, CAMAG) with phosphate-citrate buffer (6 g/L of citric acid and 10 g/L of disodium hydrogen phosphate, adjusted to pH 12 by sodium hydroxide). Therefore, except for the application zone, the plate was covered by a cut HPTLC plate silica gel 60, with the layer facing upward (Supplementary Figure S1). Then, the plates were dried at 120°C for 10 min.

### 2.3. Preparation of solutions for the enzyme, calcium chloride, standards, and samples

The digestion fluid stock solution was prepared as described by Minekus et al. (27). The pancreatin solution (200 TAME mU/μl) and the corresponding calcium chloride solution (6 pmol/μl) were prepared according to Morlock et al. (28). Monoolein, diolein, triolein, fatty acids (reference compounds were applied via overspraying to obtain the mixture on the start zone), and samples were weighed via a pipette and dissolved in *n*-hexane (all 1 mg/ml each), whereby solid fats (i.e., palm oil and coconut oil) were slightly warmed to the melting point before pipetting. All solutions were stored in solvent-tight vials in the dark at 4°C.

### 2.4. Initial triacylglycerol separation on HPTLC plate RP-18

Oil sample and FA standard solutions (10 μl/band each; 1 mg/ml) were applied as 8-mm bands, unless stated otherwise, as follows: a track distance of 10 mm, distance from the lower edge of 10 mm and left edge of 10 mm, dosage speed of 150 nl/s, filling speed of 15 μl/s, filling vacuum time of 0 s, and syringe volume of 25 μl (Automatic TLC Sampler ATS 4, CAMAG). The plate was developed with dichloromethane/acetic acid/acetone 2:4:5 (*V*/*V*/*V*) (32) in a twin-trough chamber (20 cm × 10 cm) up to 80 mm. The plate was subjected to a derivatization reagent sequence performed via dipping (immersion time 8 s, immersion speed 3 cm/s, Chromatogram Immersion Device 3, CAMAG), i.e., first in rhodamine 6G reagent solution (0.1% in ethanol) and, after plate drying and detection at FLD 366 nm (TLC Visualizer 2, CAMAG), then in copper sulfate phosphoric acid reagent (25 g copper sulfate pentahydrate in 250 ml *o*-phosphoric acid/water 4:41, *V*/*V*), followed by heating at 150°C for 20 min (TLC Plate Heater III, CAMAG) and detection at FLD 366 nm and white light illumination.

### 2.5. On-surface lipolysis and separation systems on HPTLC plate RP-18 W

The following workflow was adapted from the nanoGIT^+active^ methodology (28, 33). The application was performed as mentioned, except for a band length of 6 mm, a track distance of 9 mm, and a distance from the left edge of 14.5 mm. Reference standard mixtures were applied via overspraying. Since the previous solutions were in *n*-hexane, the cleaning unit of the ATS 4 had to be rinsed with bi-distilled water once and the syringe twice before pancreatin application. The applied sample bands were first oversprayed with pancreatin solution (5 μl/band, 200 TAME mU/μl) and then with calcium chloride solution (1 μl/band, 6 pmol/μl) using a dosage speed of 50 nl/s and a filling speed of 8 μl/s. The application zone of the plate was wetted with 2.5 ml of 0.1 M sodium chloride solution by piezoelectrical spraying (yellow nozzle, level 6, Derivatizer), whereby the plate area for chromatographic separation was covered with a cut HPTLC plate silica gel 60 (Supplementary Figure S2A) (33) to avoid the salt load on this adsorbent area and ensure good zone resolution during the later separation. This plate package was incubated at 37°C in a humid plastic box (26.5 × 16 × 10 cm, ABM, Wolframs-Eschenbach, Germany) for 60 min. After plate drying (120°C, 10 min), the lipids were focused twice by front elution with acetone up to 25 mm in a twin-trough chamber (10 min before, the second trough of the twin-trough chamber was filled up to half with molecular sieve 0.3 nm), and the lower part of the plate was cut off at 15 mm (Supplementary Figure S3) to reduce the influence of the pancreatin matrix on the chromatographic separation. The plate was developed from the cut edge side with either *n*-hexane/diethyl ether/formic acid 90:25:2 (*V*/*V*/*V*) (34) up to 60 mm for the separation of acylglycerols or acetonitrile/water 4:1 (*V*/*V*) up to 50 mm for the separation of FAs in the twin-trough chamber filled with molecular sieve as mentioned. Chromatograms were derivatized via the reagent sequence, whereby the cut 15-mm plate strip was stuck together with adhesive tape on the glass side of the HPTLC plate and detected as mentioned.

### 2.6. On-surface lipolysis and HPTLC×HPTLC analysis on HPTLC plate RP-18 W

As mentioned in the previous subsection, one oil sample per plate (10 cm × 10 cm) was applied as a 3-mm band, except for setting the distance from the left edge to 9 mm and using cover plates that covered everything but the applied sample band (Supplementary Figures S1B, S2B). The applied sample was treated with pancreatin and calcium chloride, wetted, incubated, focused (but no plate cut), developed two-dimensionally, detected via a bioassay as follows, or derivatized optionally via the reagent sequence, and detected as mentioned. For 2D development, the apolar mobile phase for acylglycerol separation was chosen as the first dimension, and the polar mobile phase for FAs separation as the second dimension. In between, the plate was dried at 120°C (TLC Plate Heater III) for 10 min and rotated by 90° (Supplementary Figure S4). Before the bioassay application, the plate was freed from residuals of the mobile phase via heating at 120°C for 10 min (TLC Plate Heater III) and neutralization with 2.5% sodium bicarbonate solution (2.8 ml, yellow nozzle, level 3, Derivatizer) followed by drying at 120°C for 10 min.

#### 2.6.1. Gram-negative *Aliivibrio fischeri* bioassay

The bacterial cryostock solution (200 μl) was suspended in 20 ml of medium according to DIN EN ISO 11348-1, Section 5 (35), and the cultivation was performed overnight (18–24 h) in a 100-ml baffled flask in room temperature by shaking at 120 rpm (KMCO2, Edmund Bühler, Hechingen, Germany). As soon as the culture showed brilliant turquoise bioluminescence when shaken in the dark, it was ready for use. The bacterial suspension was piezoelectrically sprayed onto the plate (3.5 ml, blue nozzle, level 6, Derivatizer) (36, 37), and the instant bioluminescence was recorded from the wet plate over a 30-min period (time interval 3 min, exposure time 100 s, BioLuminizer 2, CAMAG). Antibacterials and cytotoxins were detected as dark zones, whereas metabolism-enhancing substances appeared as bright zones on the bioluminescent background, depicted as a grayscale image. The positive control was caffeine (1–7 μl/band, 1 mg/ml in methanol).

#### 2.6.2. Gram-positive *Bacillus subtilis* bioassay

The bacterial stock solution (80 μl) was suspended in 20 ml of Müller–Hinton broth and incubated overnight at 37°C and 120 rpm. The culture was ready to use at an optical density measured at 600 nm (OD_600_) between 0.7 and 1.1 (Spectrophotometer M501, CamSpec, Garforth, UK). The bacterial suspension was piezoelectrically sprayed onto the plate (3.0 ml, red nozzle, level 6, Derivatizer) and incubated at 37°C for 2 h in a humid plastic box (38). Subsequently, the plate was sprayed with a 0.2% phosphate-buffered saline MTT solution (0.75 ml, blue nozzle, level 6, Derivatizer), incubated for 5 h (until an appropriate purple plate background coloring was achieved), and heated at 50°C for 10 min (TLC Plate Heater III). The positive control was tetracycline (1–7 μl/band, 10 ng/μl in ethanol). Antibacterials and cytotoxins appeared colorless (i.e., white) on a formazan-purple plate background under white light illumination.

#### 2.6.3. Planar SOS-Umu-C genotoxicity bioassay

The *S. typhimurium* TA1535/pSK1002 bacterial cryostock (25 μl) was suspended in 35 ml of LB medium (20 g/L), containing 0.1063 mg/ml of ampicillin sodium salt and 1 mg/ml of glucose, and cultivated at 37°C in a 125-ml plastic baffled flask with an aeration filter at 120 rpm for 16 h. The culture was 1:10 diluted to adjust to an OD_660_ of 0.2. The bacterial suspension was piezoelectrically sprayed onto the plate (2.8 ml, red nozzle, level 6, Derivatizer). After incubation at 37°C in a humid plastic box for 3 h, the plate was dried for 4 min in a cold air stream. The 4-methylumbelliferyl-β-d-galactopyranoside substrate (2 mg in 100 μl of dimethylsulfoxide added to 3 ml of phosphate-citrate buffer of pH 12) was piezoelectrically sprayed onto the plate (2.5 ml yellow nozzle, level 3, Derivatizer), followed by incubation at 37°C for 1 h. 4-Nitroquinoline-1-oxide (0.2–1.0 μl/band, 1 μg/ml in methanol) was used as a positive control. Genotoxins appeared as 4-methylumbelliferone-blue fluorescent zones on a dark bluish background at FLD 366 nm.

#### 2.6.4. HRMS/MS recording of active substance zones

After the bioassay, HPTLC–UV/Vis/FLD–bioassay–heart cut–RP-HPLC–DAD–HESI-HRMS/MS (39) equipped with an autoTLC interface (40) was used to analyze zones of interest directly from the bioautogram. HRMS/MS signals were recorded via the polarity switching full-scan data-dependent MS2 (ddMS2) mode. Ionization settings were equal for all MS acquisition methods: sheath gas of 20 AU, aux gas of 10 AU, a spray voltage of 3.5 kV, capillary temperature of 320°C, probe heater temperature of 350°C, and S-lens RF level 50 AU. The full-scan settings were a mass range of *m*/*z* 100–1,100, a resolving power of 70,000 (at *m*/*z* 200, full width at half-maximum, FWHM), and automatic gain control (AGC) target 3e6. Fragmentation scans followed in Top5 ddMS2 acquisition mode at a mass range of *m*/*z* 80–1,000, resolution of 17,500 FWHM, AGC target 1e6, and stepped normalized collision energies of 20, 40, and 60 eV. The HRMS/MS fragmentation data were optionally evaluated. Substances were eluted from the plate using methanol/water 1:1 (*V*/*V*). During the study, the binary pump (HPG-3200SD) of the Dionex Ultimate HPLC system (Dionex Softron, Germering, Germany) was changed to a quaternary pump (LPG-3400RS), which caused a retention time shift.

## 3. Results and discussion

### 3.1. Development of the on-surface lipolysis and both orthogonal one-dimensional separations

**Figure 1:**
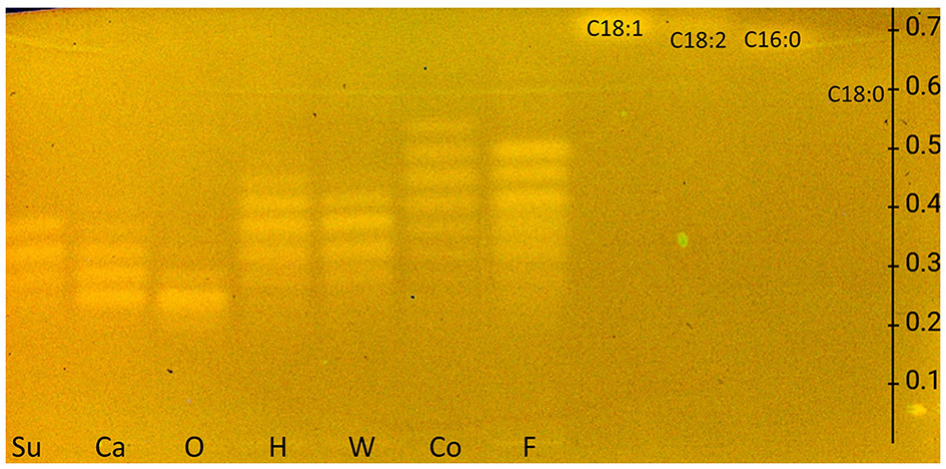
HPTLC RP-18 chromatogram of triacylglycerols in sunflower (**Su**), canola (**Ca**), olive (**O**), hemp (**H**), walnut (**W**), coconut (**Co**), and flaxseed (**F**) oils and fatty acid standards of oleic acid (C18:1), linoleic acid (C18:2), palmitic acid (C16:0), and stearic acid (C18:0), all 10 μg/band each, developed with dichloromethane/acetic acid/acetone 2:4:5 (*V*/*V*/*V*) up to 80 mm, detected at FLD 366 nm after the application of the rhodamine 6G reagent and copper sulfate phosphoric acid reagent (for comparison in Supplementary Figure S5, chromatogram with rhodamine 6G reagent only).

The European Pharmacopeia Chapter 2.3.2 method on HPTLC RP-18 plates (32) was tested first and extended to include FAs. Differently, the plate was derivatized with the rhodamine 6G reagent. The resulting FLD 366 nm chromatogram showed bands, although weak, only for FAs but not for TAGs (Supplementary Figure S5). Therefore, by exploiting the reagent sequence technique, the plate was subsequently derivatized with copper sulfate phosphoric acid reagent, and in the study, both TAG and FA zones appeared in the HPTLC RP-18 chromatogram at FLD 366 nm (Figure 1), but no charring reaction could be observed on the HPTLC RP-18 plate at white light illumination as intended for this reagent. The currently revealed fluorescence of the rhodamine 6G reagent was explained by the pH dependence of the rhodamine 6G fluorescence and the needed acidic pH for proper visualization, here provided via the copper sulfate phosphoric acid reagent (36).

Next, the plate type had to be changed from RP-18 to a wettable reversed-phase (RP-18 W), and thus, the mobile phase system had to be changed as well since the desired aim for this study was on-surface digestion via the nanoGIT method (28). Before on-surface digestion, the intrinsic acidic pH of the RP-18 W plate (ca. pH 4.2) needed to be neutralized in the application zone with a phosphate-citrate buffer of pH 12. Unfortunately, the buffer salts interfered with derivatization via the rhodamine 6G reagent (Supplementary Figure S6); therefore, everything except the application zone had to be covered as narrowly as possible (Supplementary Figure S1). Unification of the wetting and neutralization processes (after the application) to only one neutralizing wetting step is recommended, as we established in another study. The pancreatin matrix interfered during development by causing a retardation shift in contrast to the reference standards. Thus, it had to be removed by focusing the lipids twice by front-elution with acetone after the lipolysis and cutting off the lower plate part containing the remaining pancreatin matrix. Since the focusing result with acetone was strongly dependent on the relative humidity of the laboratory environment, the second trough was half-filled with a molecular sieve of 0.3 nm within 10 min prior to focusing. When a dry environment (< 15% relative humidity) in the twin-trough chamber was reached, acetone was filled in the opposite trough, and the plate was placed inside as fast as possible for development. The dry conditions during focusing as well as the (second) polar mobile phase development showed reproducibly good zone resolutions.

**Figure 2:**
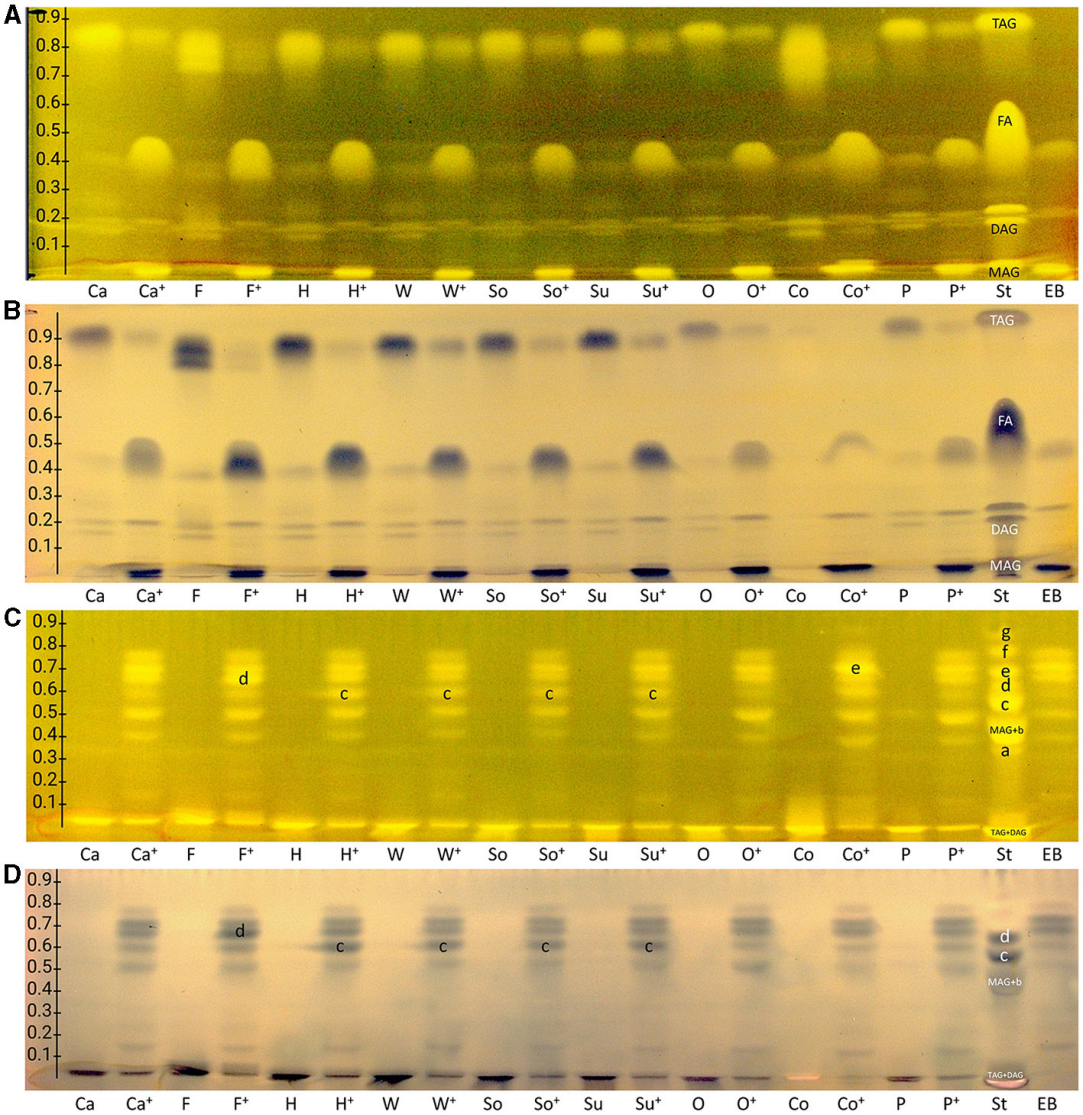
nanoGIT–HPTLC RP-18 W chromatograms of **(A, B)** acylglycerols and **(C, D)** fatty acids (**FAs**) in canola (**Ca**), flaxseed (**F**), hemp (**H**), walnut (**W**), soybean (**So**), sunflower (**Su**), olive (**O**), coconut (**Co**), and palm (**P**) oil (all 10 μg/band each) before and after (^**+**^) lipolysis via overspraying of pancreatin (each 1 TAME U/band), focused twice with acetone and, after plate cut off at 15 mm, developed (from the cut edge) with **(A, B)** *n*-hexane/diethyl ether/formic acid 90:25:2 (*V*/*V*/*V*) up to 60 mm, or **(C, D)** acetonitrile/water 4:1 (*V*/*V*, with molecular sieve) up to 50 mm, derivatized as reagent sequence on the same plate **(A, C)** first with rhodamine 6G reagent detected at FLD 366 nm, **(B, D)** followed by copper sulfate phosphoric acid reagent detected at white light illumination (in remission); for comparison, standards (**St**) triolein (**TAG**), diolein (**DAG**), monoolein (**MAG**), stearic acid (C18:0, **a**), palmitic acid (C16:0, **b**), oleic acid (C18:1, **b**), linoleic acid (C18:2, **c**), myristic acid (C14:0, **c**), linolenic acid (C18:3, **d**), lauric acid (C12:0, **e**), capric acid (C10:0, **f**), and caprylic acid (C8:0, **g**) and enzyme blank (**EB**, 1 TAME U/band of pancreatin).

Due to the amphiphilic properties of the RP-18 W phase (apolar C18 chains and residual silanol groups), two orthogonal mobile phase systems were developed, i.e., one apolar to separate acylglycerols and one polar to separate FAs. Both resulting nanoGIT–HPTLC RP-18 W chromatograms (Figures 2A, C) proved the orthogonality of the mobile phases. Surprisingly, the derivatization with rhodamine 6G directly showed all lipophilic compounds. This variation in the fluorescence response was explained by different initial plate pHs and proven by additional experiments, in which the rhodamine 6G fluorescence showed a strong plate batch dependence due to different plate pHs. Fortunately, the current plate batch pH supported the required acidic milieu for the rhodamine 6G fluorescence (41). To exploit a reagent sequence, the subsequent derivatization of the same plate with copper sulfate phosphoric acid reagent (Figures 2B, D) led to a charring reaction of all unsaturated lipophilic compounds detected as black zones. The combination of both derivatization reagents on the same plate surface made it possible to first visualize all lipophilic compounds and then differentiate saturated and unsaturated zones.

As observed for the standard mixture (Figures 2A, B, **St**), the separation of acylglycerols according to polarity in TAGs, DAGs, and MAGs was successful, but all the reference FAs were eluting as one unseparated diffuse zone (*hR*_F_ 37–57). This system also allowed the separation of both DAG isomers (*hR*_F_ 19 and 26) and TAG isomers, as observed for flaxseed oil (**F**, *hR*_F_ 80 and 86). Comparing the side-by-side separated undigested and digested (^**+**^) samples, a massive increase in the FAs and decrease in the TAGs amount/signal was observed, indicating the successful simulation of the lipolysis. The undigested samples showed two bands at the DAG zone; however, after the lipolysis, only one band remained, which was caused by the pancreatin enzyme blank (**EB**). Two further interferences were observed from the pancreatin blank, one intense zone was just below the MAGs, and another weaker zone was at the FAs position (assigned to triterpenoid acids as discussed later), which complicated the evaluation of those in the sample. Nevertheless, the comparison of both derivatization reagents supported the literature-known oil composition of all samples. Oils with a variety of FAs, such as flaxseed, hemp, and coconut oils, showed broader TAG and FA zones, whereas olive oil (mainly containing C18:1) showed comparatively compact zones. Concentrating on the unsaturated FAs in the copper sulfate phosphoric acid chromatogram (Figure 2B), the most intense zones for flaxseed oil (mainly PUFAs) and, in contrast, almost no zones for coconut oil (containing comparatively much more SFAs) confirmed the oil compositions as well.

**Figure 3:**
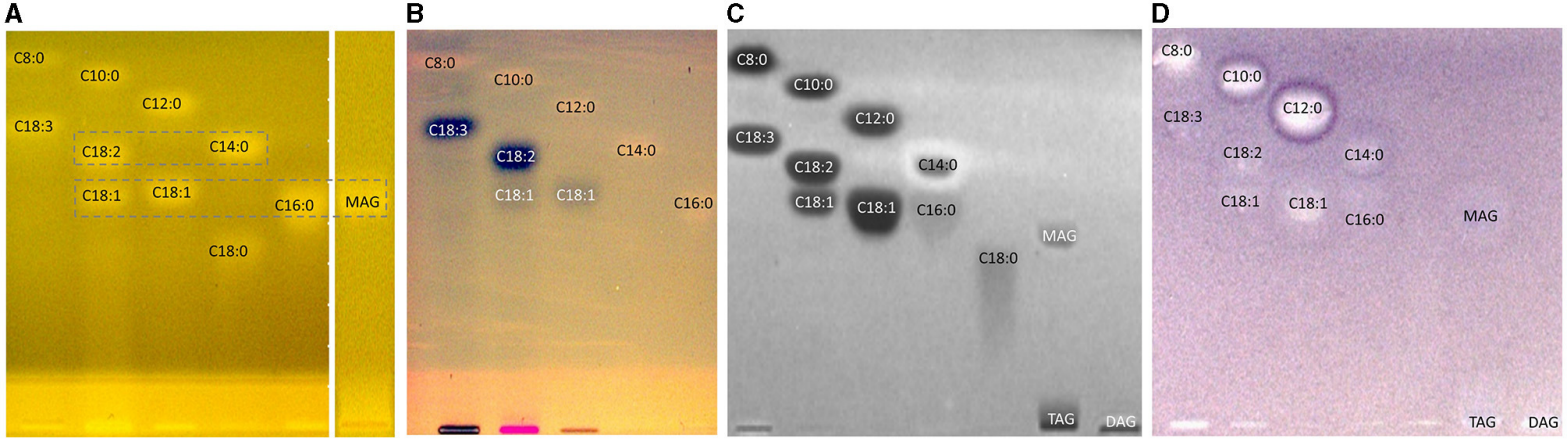
HPTLC RP-18 W chromatograms **(A, B)** and bioautograms **(C, D)** of fatty acids (**C8:0**–**C18:3**) and acylglycerols (**MAG**, **TAG**, and **DAG**), 10 μg/band each, separated with acetonitrile/water 4:1 (*V*/*V*, with molecular sieve) up to 50 mm, detected **(A)** at FLD 366 nm after derivatization with rhodamine 6G reagent, and a white light illumination (in remission) after **(B)** derivatization as reagent sequence first with rhodamine 6G reagent followed by copper sulfate phosphoric acid reagent, **(C)** Gram-negative *Aliivibrio fischeri* bioassay (bioluminescence depicted as a greyscale image), and **(D)** Gram-positive *Bacillus subtilis* bioassay.

The orthogonal selectivity selected for the separation of FAs according to lipophilicity showed a successful qualitative separation of nearly all FA reference standards (Figures 2C, D, **St**). In this system, TAGs and DAGs were retained at the application zone, whereas the MAGs were eluted. Due to their similar lipophilicity, C18:1, C16:0, and MAGs co-eluted as well as C18:2 and C14:0, which was proven in another experiment (Figure 3, framed). In the nanoGIT–HPTLC RP-18 W chromatogram (Figures 2C, D), the undigested samples did not show any noticeable FA zones, but the digested samples did. Thus, the lipolysis of TAGs into FAs was successful. High FA amounts as in the reference track (Figures 2C, D, **St**) and pancreatin matrix effects on the sample tracks led to a retardation shift; thus, the zone matching between the samples and reference compounds was challenging but nevertheless possible. With the aid of the copper sulfate phosphoric acid reagent chromatogram, C18:2 and C18:3 could be identified as intense black zones. The rhodamine 6G reagent chromatogram helped identify C8:0, C10:0, and C12:0. Due to this assigned pattern of the FAs and the literature data (37, 38), the FAs in the samples could be identified successfully via pattern recognition based on their main components. Intense zones for C18:3 (zone **d**) in flaxseed oil, C18:2 (zone **c**) in hemp, walnut, soybean, and sunflower oils, and C14:0 (zone **e**) in coconut oil were determined after digestion via pancreatin (Figures 2C, D).

**Figure 4:**
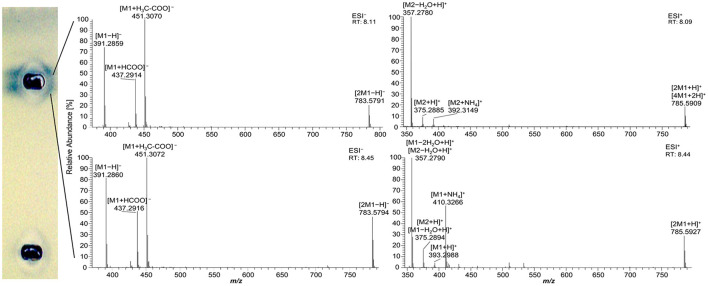
High-resolution mass spectra and corresponding ion species in the negative and positive ionization mode after nanoGIT–HPTLC –Vis/FLD–heart cut–RP-HPLC–DAD–HESI-HRMS/MS analysis of pancreatin developed with acetonitrile/water 4:1 (*V*/*V*) and molecular sieve (3 Å) up to 50 mm. **M1** = ursodeoxycholic acid, hyodeoxycholic acid, (cheno)deoxycholic acid, **M2** = cholenic acid; after the transfer of the interesting zones to the HRMS, the stamped plate was derivatized using the copper sulfate phosphoric acid reagent to check whether the elution head was properly positioned on the zones of interest.

As mentioned for the separation of acylglycerols, polar impurities of the pancreatin co-eluting with the FAs hindered their assignment and could also lead to false-positive interpretations. Using automated heart-cut elution of the interesting zones to RP-HPLC–DAD–HESI-HRMS/MS (39), these impurities were assigned as the bile acids ursodeoxycholic acid (UDCA), hyodeoxycholic acid (HDCA), (cheno)deoxycholic acid (CDCA/DCA), and cholenic acid (Figure 4 and Supplementary Table S2). The isomers UDCA, HDCA, and (C)DCA were identified in the negative ionization mode (HESI^−^) in two separate peaks at retention times of 8.11 min and 8.45 min with [M–H]^−^ at *m/z* 391.2858 and 391.2860, respectively. The HESI^−^ and respective positive ionization mode (HESI^+^) revealed the presence of their dimers ([2M–H]^−^ at *m/z* 783.5791 and [2M+H]^+^ at *m/z* 785.5927, Figure 4), identified via fragmentation (Supplementary Figure S7), as well as their tetramer ([4M+2H]^+^ at *m/z* 785.5909 with its corresponding isotopic pattern, Supplementary Figure S8). Cholenic acid could only be assigned via HESI^+^ as [M+H]^+^ at *m*/*z* 375.2885. Since bile acids also show antibacterial properties (40), false-positive results should be considered and, when necessary, confirmed/excluded via HRMS. Fortunately, the following comprehensive separation system solved this coelution issue.

### 3.2. Development and proof of the nanoGIT–HPTLC×HPTLC–FLD on RP-18 W plates

**Figure 5:**
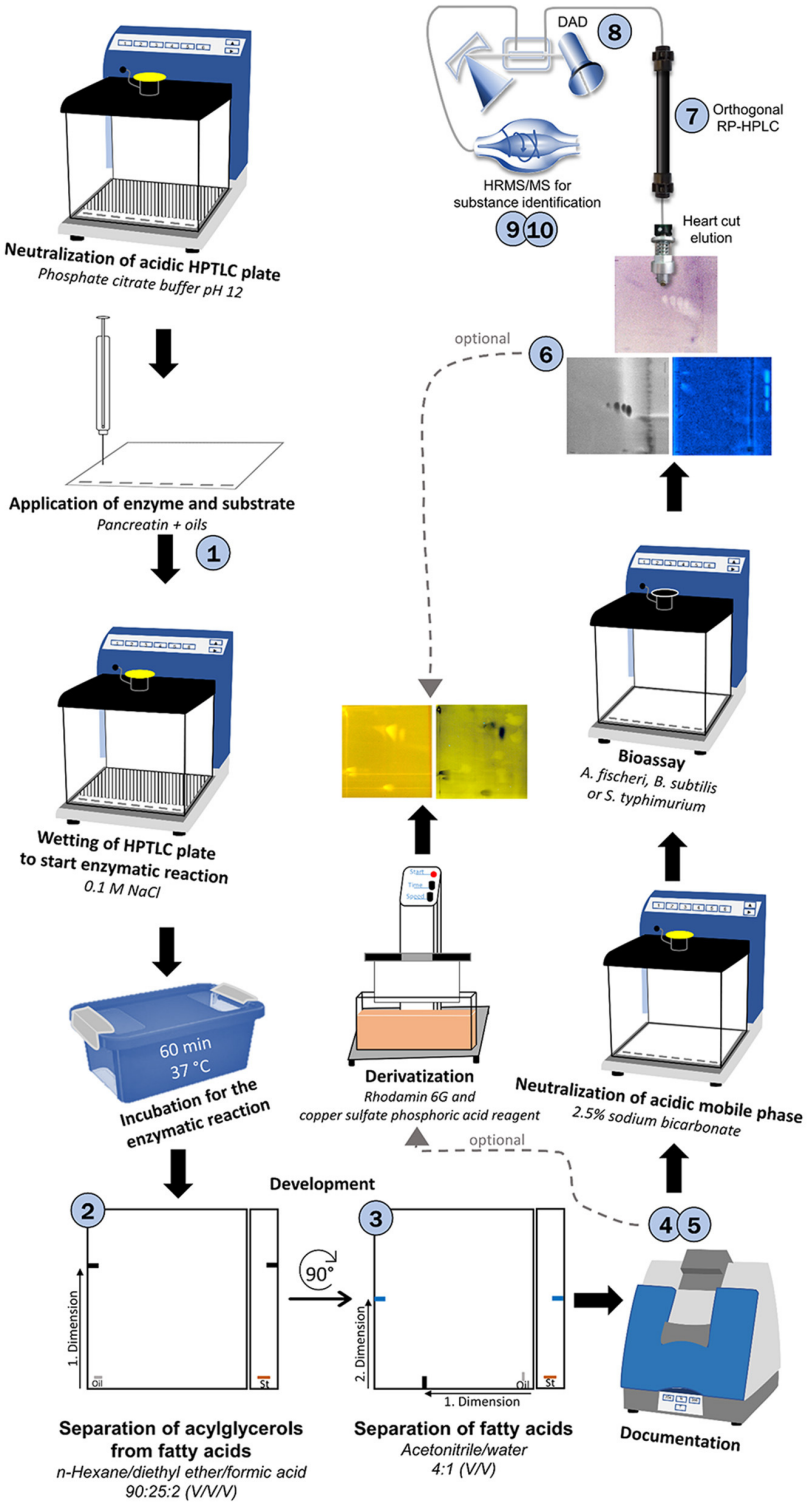
Schematic workflow of the developed 10D hyphenation, combining lipolysis with analysis and effect-detection on the same surface: nanoGIT–HPTLC×HPTLC–Vis/FLD–bioassay–heart cut–RP-HPLC–DAD–HESI-HRMS/MS.

**Figure 6:**
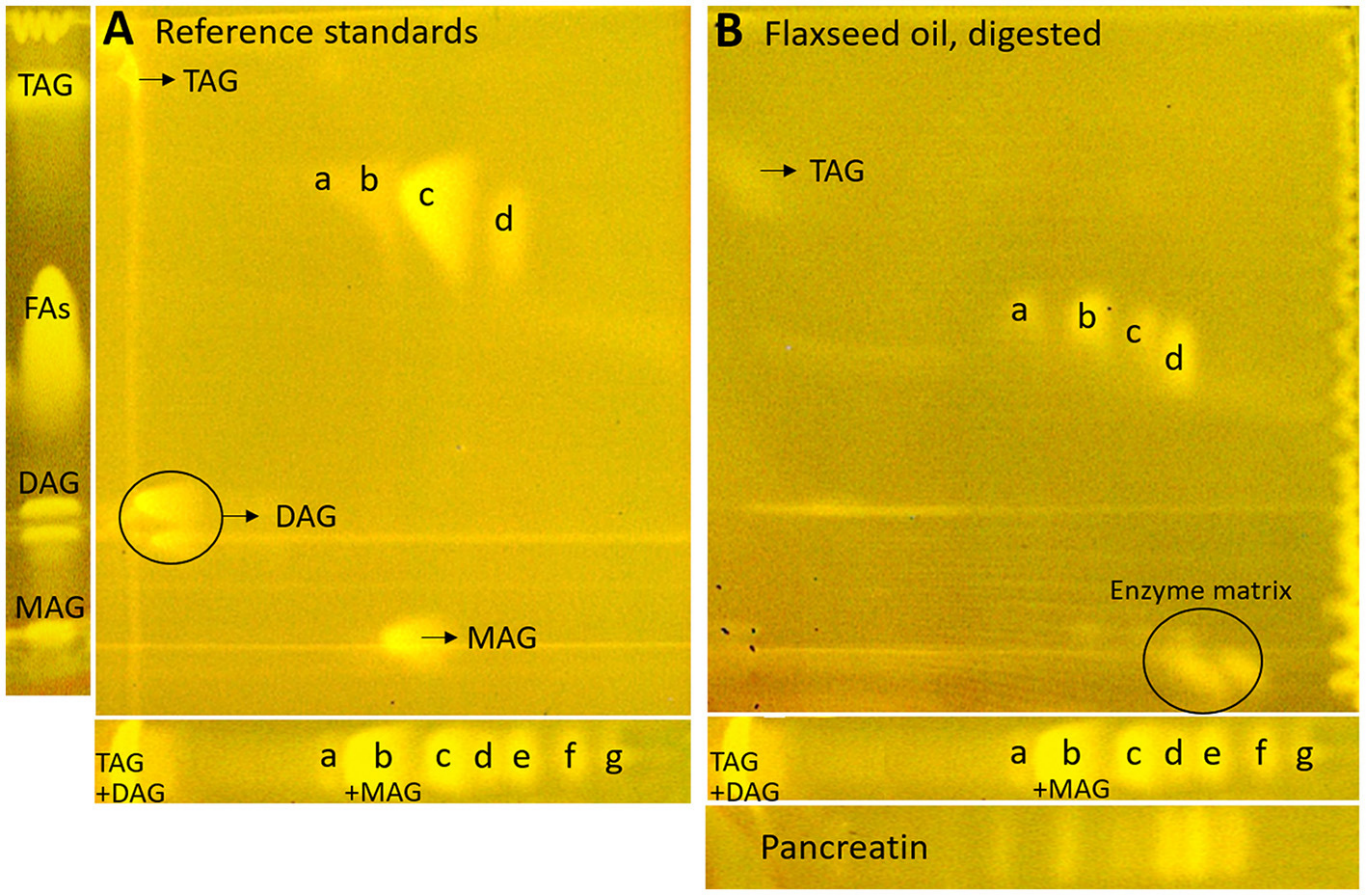
nanoGIT–HPTLC×HPTLC–FLD chromatograms of **(A)** the reference sample mixture (undigested) and **(B)** flaxseed oil (all 10 μg/band each) after lipolysis via overspraying of pancreatin (1 TAME U/band) on HPTLC RP-18 W plate, focused twice with acetone and, after a plate cut off at 15 mm, developed (from the cut edge) first with *n*-hexane/diethyl ether/formic acid 90:25:2 (*V*/*V*/*V*) up to 60 mm and, after 90°-plate turn, then with acetonitrile/water 4:1 (*V*/*V*, with a molecular sieve) up to 50 mm, derivatized with rhodamine 6G reagent, and detected at FLD 366 nm; for comparison respective one-dimensionally separated references, i.e., acylglycerols (**MAG**, **TAG**, and **DAG**) and fatty acids (**FAs**) such as stearic acid (C18:0, **a**), palmitic acid (C16:0, **b**), oleic acid (C18:1, **b**), linoleic acid (C18:2, **c**), myristic acid (C14:0, **c**), linolenic acid (C18:3, **d**), lauric acid (C12:0, **e**), capric acid (C10:0, **f**), and caprylic acid (C8:0, **g**), all 10 μg/band each.

The one-dimensional separation systems showed some limits, such as interferences with the pancreatin used and no separation of acylglycerols and FAs at once, which could not be overcome by method optimization or modification. Hence, the combination of both orthogonal separation systems into a comprehensive HPTLC method (HPTLC×HPTLC) was evaluated (Figure 5). The normal phase separation mechanism separating according to polarity (apolar acylglycerol-separating mobile phase) was chosen to be the first dimension, whereas the reversed-phase separation mechanism separating according to lipophilicity (polar FA-separating mobile phase) was selected as the second dimension. The orthogonality was given by the very different selectivity of the first dimension in contrast to the second dimension. The orthogonal separation was first tested with reference standards (Figure 6A), and successful separation of acylglycerols and FAs could be achieved, in particular the separation of the previously co-eluted MAGs and FAs. The zone assignment of the FAs in the nanoGIT–HPTLC×HPTLC–FLD chromatogram was more difficult than via one-dimensional separation. Using the co-development of reference standards for each dimension on a separate plate (Supplementary Figure S4), a retardation shift could be observed for the FA zones. Since both mobile phases were prone to changes in relative humidity, the co-development of reference standards on the same plate was recommended to verify a retardation shift and proper assignment. The dominance of the C18:2 and C18:3 fatty acids (zones **c** and **d**, respectively) was also helpful for proper assignment.

Next, the on-surface digestion of an oil sample and the subsequent lipid separation of its lipolysis products on the same surface were demonstrated (Figure 6B). Therefore, flaxseed oil rich in C18:3 and C18:2 was selected for the proof of principle. A retardation shift was observed between reference standards (Figure 6A) and samples (Figure 6B). Using automated heart cuts to RP-HPLC–DAD–HESI-HRMS/MS (39), the highest eluting FAs (Figure 6B, zone **d**) were identified as C18:3, C18:2, C16:0, and C18:1 (Supplementary Figure S9, zone **d**, Supplementary Table S3). The most intense signal for this zone was from C18:3. Since it was stamped perpendicular to the band due to an accidentally 90°-rotated plate (positioned incorrectly) in the autoTLC interface (42), several FA signals were derived from and assigned to the neighboring bands. Some additional FAs were identified that could not be associated with the flaxseed oil sample: in zones **c** and **d**, oxidized C9:0 and oxidized C12:1 were identified, which could be explained as degradation products of linoleic acid and linolenic acid, respectively. In zone **d**, C14:0, and zone **e**, C10:0, C11:0, and C12:0 were found. No fragmentation pattern was evident via HRMS/MS recording. The previously interfering pancreatin matrix was presently successfully separated from the FAs since most pancreatin impurities did not elute in the first dimension (in contrast, the FAs did) but did elute first in the second dimension. Thus, the FAs could be identified easily, in contrast to the one-dimensional separation of the reference standards (Figure 6B). Additionally, the eluted FA zones were fully separated from the bile acids, and their mass signals were not detected in the HRMS spectra anymore. By doing so, the nanoGIT–HPTLC×HPTLC–FLD method was proven to be successful in its application to real-life samples and in the detailed study of the lipolysis of complex samples. The whole sample was studied in all aspects on the same surface, and no sample part was lost.

### 3.3. Antibacterial profiles via nanoGIT–HPTLC×HPTLC–vis/FLD–bioassay–heart cut–RP-HPLC–DAD–HESI-HRMS/MS

**Figure 7:**
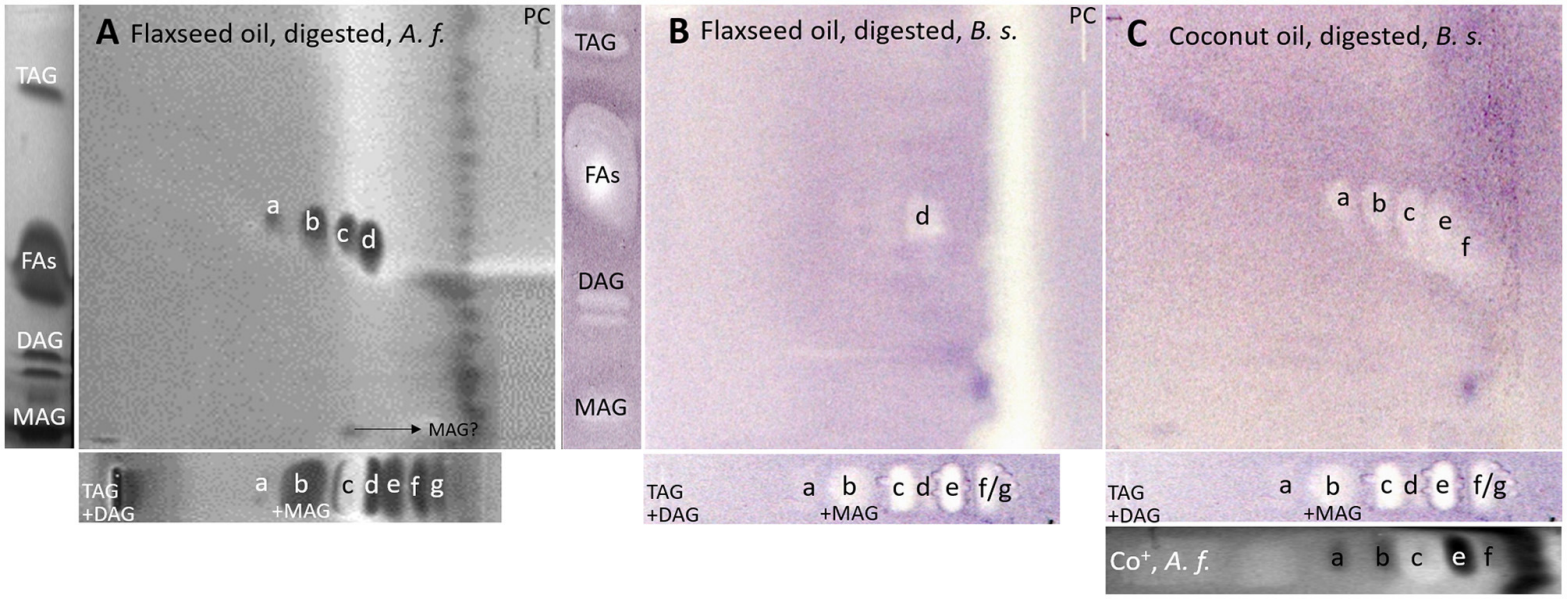
nanoGIT–HPTLC×HPTLC–bacterial bioassay–FLD/bioluminescence: Gram-negative *Aliivibrio fischeri* and Gram-positive *Bacillus subtilis* bioautograms showing the antibacterial activity (dark or colorless zones, respectively) of **(A, B)** digested flaxseed oil and **(C)** digested coconut oil (10 μg/band each) analyzed as in Figure 5, detected **(A)** via the bioluminescence (depicted as a grayscale image) after the *A. fischeri* bioassay and **(B, C)** at white light illumination (in remission) after the *B. subtilis* bioassay; positive controls (**PCs**) were 10–70 μg/band caffeine for *A. fischeri* bioassay or 10–70 ng/band tetracycline for *Bacillus subtilis* bioassay.

After a successful proof of principle and implementation of the nanoGIT–HPTLC×HPTLC–FLD hyphenation, it was extended to bioassays to evaluate the antibacterial activity of the lipolysis products of digested flaxseed oil (Figures 7A, B) and coconut oil (Figure 7C) against Gram-negative *A. fischeri* and Gram-positive *B. subtilis* bacteria. The *A. fischeri* bioautogram revealed antibacterial effects for all seven FA reference standard zones as well as for the MAG, DAG (both isomers of diolein), and TAG reference standard zones (Figure 7A). In the *B*. *subtilis* bioautogram, the antibacterial detection was comparable, apart from the weaker response for two FA reference zones (**d** and **g**, Figure 7B), which was proven and confirmed in another experiment (Figure 5). These findings of antibacterial activity were consistent with the literature, which confirmed the antibacterial effect of FAs and MAGs (8, 41, 43, 44) and DAGs (9) against Gram-negative and Gram-positive bacteria. Usually, no antibacterial effect for TAGs (three ester groups but optionally double bonds) would be expected due to the lack of reactive functional groups (41, 45). However, the studied TAG, DAG, and MAG had one double bond in each acyl chain, which could induce a genotoxic or cytotoxic effect, as discussed later.

A separate study of all reference standards (Figure 3) showed in more detail the differences in their antibacterial effects against both Gram-negative and Gram-positive bacteria. The FAs C18:0 and C16:0 showed only a very light antibacterial response, whereas a metabolism-enhancing effect (detected as a halo surrounding an antibacterial effect in the center) was detected for C14:0. If co-eluted with C18:2 as in the standard track, this enhancing effect was weakened since the antibacterial effect of C18:2 was stronger (Figure 7, zone **c**). Compared to previous bioautograms on HPTLC plates silica gel 60 (39, 46), C16:0 showed no metabolism-enhancing effect on RP-18 W plates, which was explained by the doubled amount (10 μg/band vs. 5 μg/band) since such enhancing effects are dose-dependent and, in addition, also time-dependent (bioluminescence images monitored for 30 min). The antibacterial response for C8:0–12:0 was very intense against both Gram-negative *A. fischeri* and Gram-positive *B*. *subtilis* bacteria (Figure 3). In the *A*. *fischeri* bioautogram, a strong antibacterial effect of unsaturated FAs (C18:1–C18:3) against *A*. *fischeri* was observed, whereas an increase in the antibacterial effect with increasing double bonds was not observed. In the *B*. *subtilis* bioautogram, the antibacterial effect of unsaturated FAs against Gram-positive bacteria was weaker, which was directly evident since the same reference standard amounts were applied. Further research is needed to understand the mechanism of the observed biological responses of the FAs and acylglycerols. On the one hand, the biological response may derive from the acid head group and/or altered membrane permeability and thus be an antibacterial effect (as one example of the many different antibacterial mechanisms). On the other hand, the biological effect may derive from trace impurities (e.g., co-eluting epoxidized longer-chain fatty acids, Figure 3) in the reference standards (only up to 99% pure) or oxidative degradation and thus be a cytotoxic effect. Unfortunately, the separation power of HPTLC is too weak to chromatographically differentiate all of them. Nevertheless, the powerful hyphenation with the bioassay provides the first evidence of harmful compounds present.

**Figure 8:**
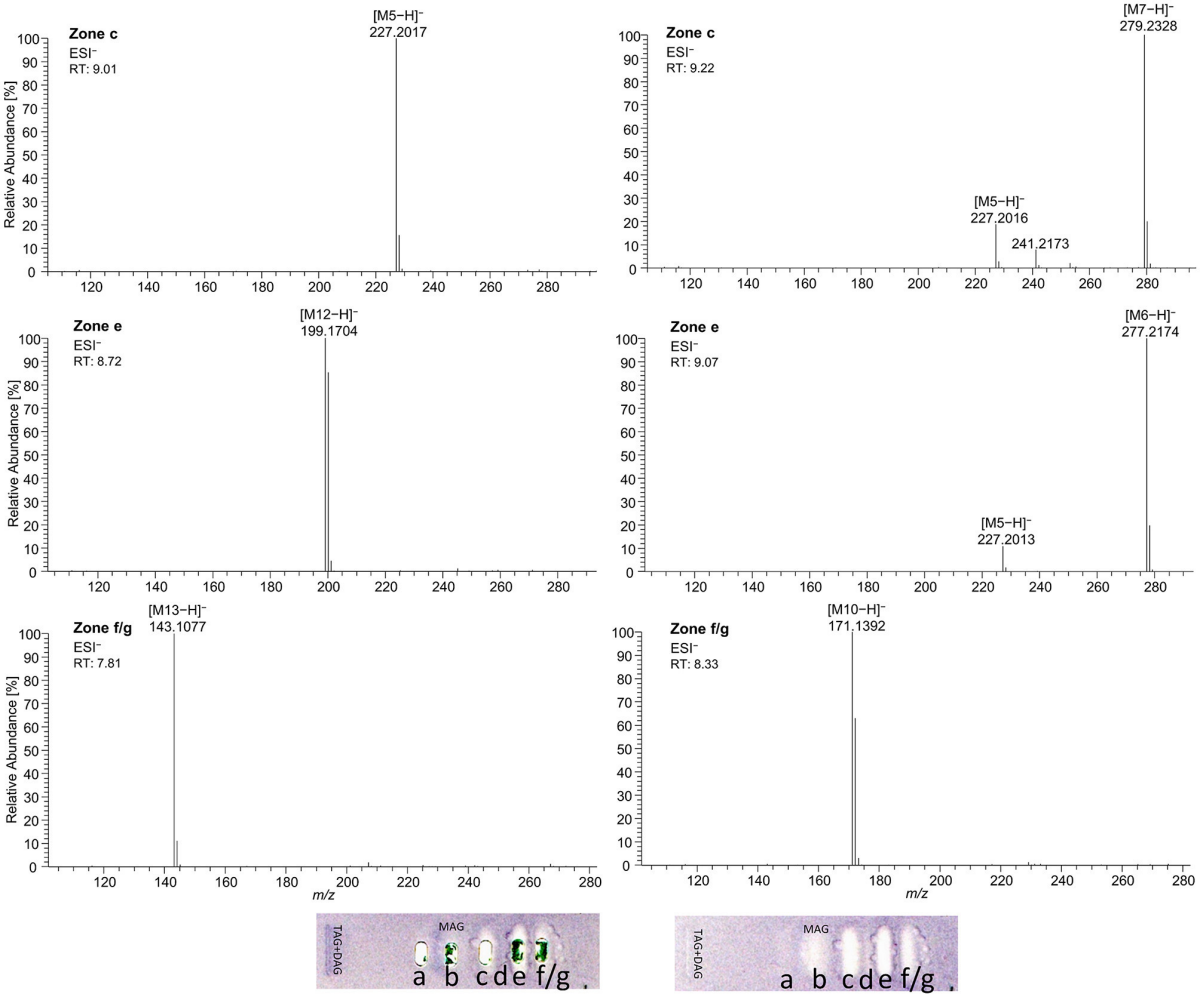
High-resolution mass spectra and corresponding ion species in the negative ionization mode after HPTLC–Vis–bioassay–heart cut–RP-HPLC–DAD–HESI-HRMS/MS analysis on HPTLC RP-18 W plates of all identified fatty acids of the standard reference track after the *Bacillus subtilis* bioassay, which was developed with acetonitrile/water 4:1 (*V*/*V*, with molecular sieve) up to 50 mm; **M5** = C14:0, **M6** = C18:3, **M7** = C18:2, **M10** = C10:0, **M12** = C12:0, and **M13** = C8:0.

Using automated heart cuts of the interesting zones to RP-HPLC–DAD–HESI-HRMS/MS, the Gram-positive antibacterial zones of the reference standard track (Figure 7B) could be identified as the corresponding FAs (Figure 8, Supplementary Table S4). The assignments for zones **a** (C18:0) and **b** (C16:0/C18:1) were reached through pattern recognition. The latter assignments were more challenging since these FAs can also be HRMS system signals, which must be excluded first. Zones **c** and **e** were the most intense, containing co-eluting C14:0/C18:2 and C12:0/C18:3 (from adjacent zone **d**), respectively. Zone **d** (C18:3) was too close to the surrounding zones for an elution head-based analysis (too narrow for an additional elution head imprint). The C8:0 (zone **g**) was not separated in the 2D bioautogram but co-eluted with the C10:0 (zone **f**).

Considering the information obtained about the antibacterial behavior of the reference standards, the assignment of the lipolysis products of flaxseed oil was possible. Flaxseed oil, which mainly consists of C18:3, C18:1, and C18:2 and small amounts of C16:0 and C18:0 (38), showed after on-surface digestion and effect-directed analysis of four zones in the 2D *A*. *fischeri* bioautogram but only one zone in the 2D *B*. *subtilis* bioautogram (Figures 7A, B). The four zones in the 2D *A*. *fischeri* bioautogram were identified as C18:0 (zone **a**), C18:1/C16:0 (zone **b**), C18:2 (zone **c**), and C18:3 (zone **d**) in comparison to corresponding reference standards. No metabolism-enhancing effect was detected, and thus, the presence of C14:0 was excluded, which could have co-eluted with C18:2. The antibacterial zone **d** in the *B*. *subtilis* bioautogram (Figure 7B) was not so clear in the assignment, and thus identified via HRMS as C18:3 ([M–H]^−^, *m/z* 277.2175, Δ ppm −0.71) and *trans*-4,5-epoxy-(*E*)-2-decenal (C_10_H_16_O_2_, [M–H]^−^, *m/z* 167.1078, Δ ppm −0.28). The latter is a typical genotoxic marker of linoleic acid oxidation (47–49).

**Figure 9:**
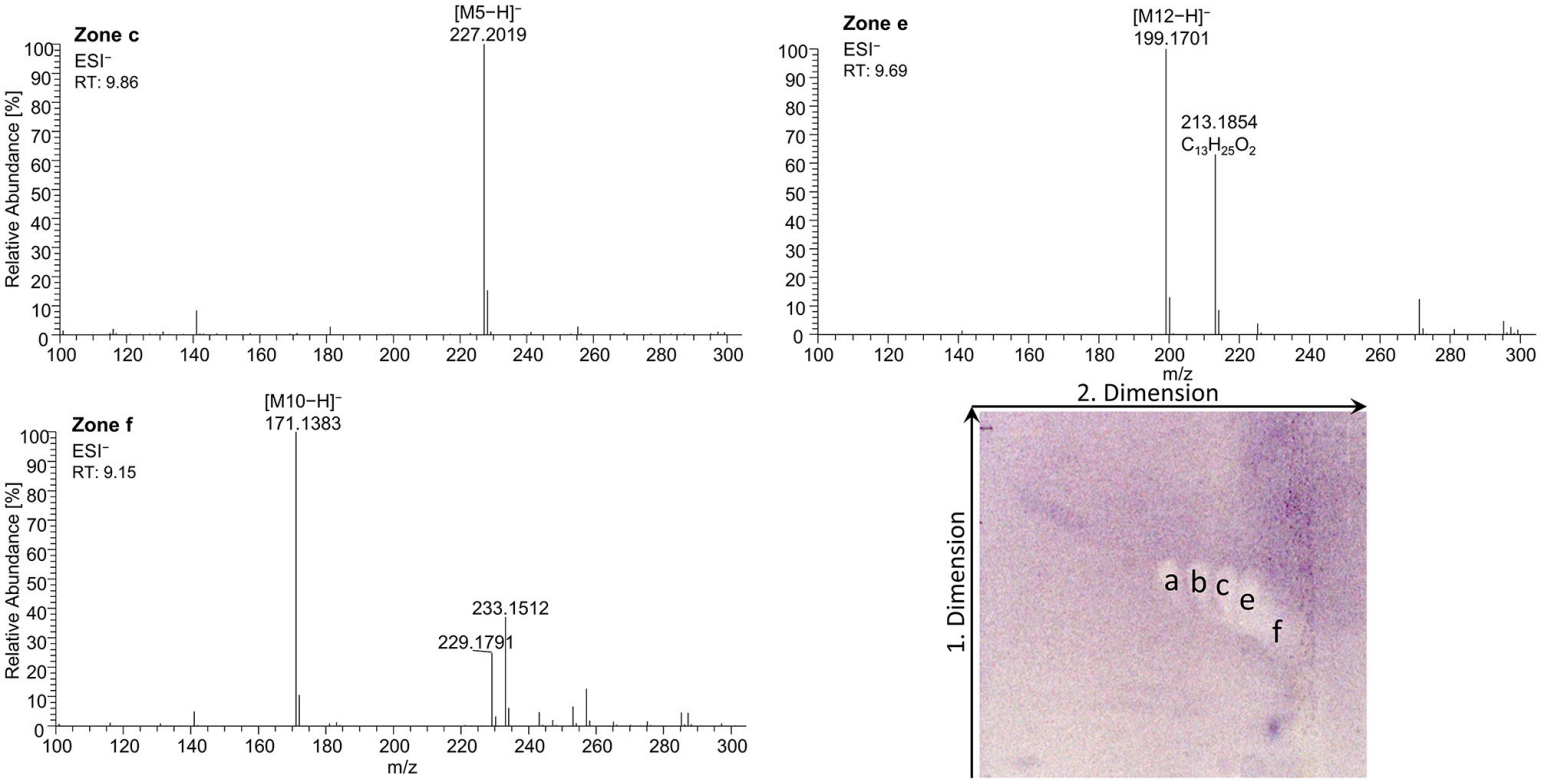
High-resolution mass spectra and corresponding ion species in the negative ionization mode after the nanoGIT–HPTLC×HPTLC–Vis–bioassay–heart cut–RP-HPLC–DAD–HESI-HRMS/MS analysis of all identified fatty acids of digested coconut oil after the *Bacillus subtilis* bioassay on HPTLC RP-18 W plates, where the plates were focused twice with acetone and cut at 15 mm, developed (from the cut edge) first with *n*-hexane/diethyl ether/formic acid 90:25:2 (*V*/*V*/*V*) up to 60 mm, after 90° plate turn, and then with acetonitrile/water 4:1 (*V*/*V*, with molecular sieve) up to 50 mm; retention time shift due to HPLC pump exchange; **M5** = C14:0, **M10** = C10:0, and **M12** = C12:0.

As a further example, coconut oil was digested on-surface and analyzed for any antibacterial effects (Figure 7B). Coconut oil was selected as a quite different oil sample compared to flaxseed oil since it consists of comparatively much more SFAs of shorter chain lengths (C8:0–14:0, mostly C12:0 and C14:0) (37, 45). In contrast to the flaxseed oil (one antibacterial zone), the 2D *B*. *subtilis* bioautogram showed five antibacterial zones. Using the standard track, the FAs were identified as C18:0 (zone **a**), C18:1/C16:0 (zone **b**), C18:2/C14:0 (zone **c**), C12:0 (zone **e**), and C10:0 (zone **f**); however, C18:3 (zone **d**) was not present. The zone was heart-cut eluted to RP-HPLC–DAD–HESI-HRMS/MS (Figure 9, Supplementary Table S5) and revealed C14:0, C12:0, and C10:0 signals for the zones **c**, **e**, and **f**, respectively, but no significant signals for C18:0 and C18:1/C16:0. The *A*. *fischeri* bioautogram of the on-surface digested coconut oil (Figure 7C, Co^+^) was used to confirm zone **c** to be C14:0 via its metabolism-enhancing effect as further proof.

### 3.4. Genotoxicity profiles via nanoGIT–HPTLC×HPTLC–vis/FLD–bioassay–heart cut–RP-HPLC–DAD–HESI-HRMS/MS

**Figure 10:**
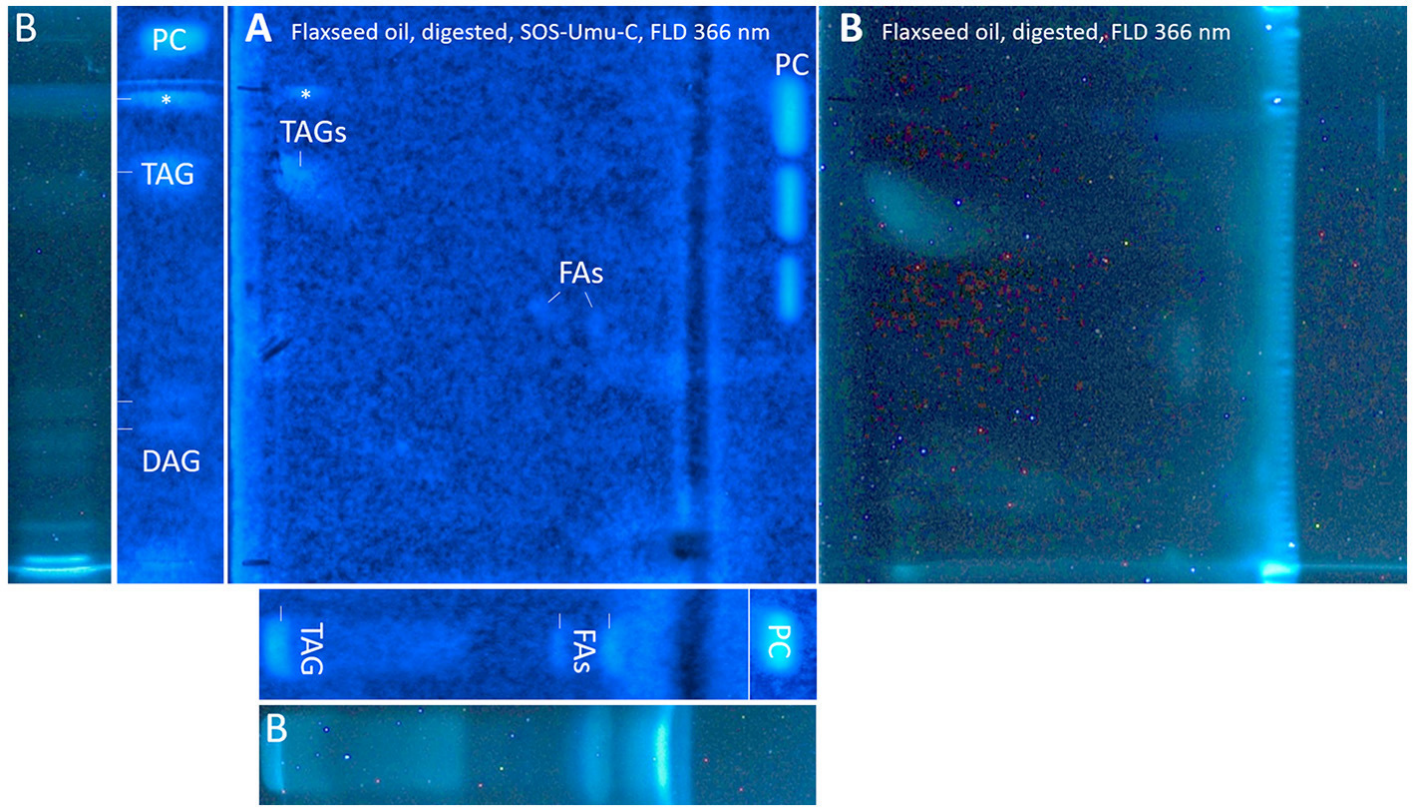
nanoGIT–HPTLC×HPTLC–FLD–SOS-Umu-C bioassay–FLD: genotoxicity bioautogram (using *Salmonella typhimurium* TA1535/pSK1002) of digested flaxseed oil (20 μg/band) analyzed as in Figure 5, detected at FLD 366 nm **(A)** after the genotoxicity bioassay, showing genotoxic (blue fluorescent) TAGs, DAGs, FAs, and an unknown apolar genotoxic substance zone (*, e.g., mineral oil aromatic hydrocarbons); **(B)** comparative chromatogram before the bioassay; 0.2–1.0 ng/band 4-nitroquinoline-1-oxide as a positive control (**PC**).

On-surface digested flaxseed oil revealed four genotoxic substance zones in the 2D bioautogram after the genotoxicity bioassay using the *S. typhimurium* TA1535/pSK1002 strain (Figure 10A). Two genotoxic substance zones did not migrate/elute at all in the second dimension, indicating apolar substances. One zone was assigned as TAGs via comparison with the standard track, and the second more apolar compound (marked^*^ close to the solvent front of the first dimension) could be a genotoxic aromatic mineral oil contaminant; however, the latter assumption still needs proof. The genotoxic effect of TAGs was explained by the epoxidized fatty acid bond in the TAG molecule. Only two weak signals for the FAs were detected in the flaxseed oil and reference standard mixture (second dimension), which were assigned to C18:2/C14:0 and C18:3/C12:0 or C10:0. The digestion of the TAGs did not eliminate genotoxicity but showed that the FAs produced have strongly different genotoxic potentials. Both FAs were not natively fluorescent, which was expected (Figure 10B); however, native blue fluorescence was observed for the genotoxic TAGs zone of flaxseed oil, which indicated any impurities, e.g., of aromatic structure, as mentioned.

For adequate signal intensity via the genotoxicity bioassay, the amount of flaxseed oil was doubled (20 μg/band). In contrast to our previous very sensitive screening method (10), the amount of oil needed to be increased 200-fold due to the (I) enzymatic metabolization with a 60-min on-surface incubation known to lead to diffusion at the application zone (33), (II) interference by the buffer salts (Supplementary Figure S6), (III) 2D separation known to cause signal loss (50) by the 2-fold diffusion of the substances (as for C18:0, Figure 3), (IV) usage of RP-18 W plates known to be possibly less sensitive in the response, though dependent on the molecule, compared to silica gel 60 plates (51, 52), and (V) purchased oils opened just before analysis (assumedly, comparatively fewer oxidized degradation products). These reasons also explained why HRMS analysis was challenging since oxidized species present at the trace level were not found. In contrast to Morlock and Meyer (19), in which a six-fold concentrated genotoxic compound zone was directly transferred to the HRMS, only one weaker genotoxic compound zone was eluted from the 2D bioautogram, passed through an HPLC column via a prior desalting unit and diode-array detector, and finally reached the HRMS. The presence of highly potent genotoxic FA in oxidized and epoxidized forms at the trace level in various plant oils (10, 49, 53), and its potential sources, such as the unsaturated FAs C18:2 (54) and C18:3 (54, 55), were already reported. If safely delivered to a healthy liver, detoxification may be expected, as was recently shown via simulated on-surface S9 liver metabolization (10, 56). Furthermore, synergistic effects can occur (57), which can be detected and studied via the latest multiplex planar assays (51).

The advantages and disadvantages of this quite new methodology against reported conventional methods (Supplementary Table S6) (58, 59), including the further ability of an effect-directed analysis after separation, strongly highlight the ability to illuminate every facet of the sample.

## 4. Conclusion

The on-surface simulated digestion on the RP-18 W plate, followed by comprehensive orthogonal HPTLC×HPTLC separation and effect-directed bioassay detection, successfully demonstrated a sustainable all-in-one digestion and analysis system. It allowed the analysis of the digestion during the intestinal phase itself and the resulting products as well as their biological effects via antibacterial and genotoxic bioassays. Since the developed method included a 2D development, the sample throughput was limited to only one sample per plate, but two sample plates could be processed at the same time with the HPTLC system used. The low solvent consumption (max. 16 ml per analysis/two plates) and rather short analysis time (5 h per analysis/two plates including bioassay and MS) endorsed the application as a multi-faceted analysis system. The developed 10D hyphenated nanoGIT–HPTLC×HPTLC–Vis/FLD–bioassay–heart cut–RP-HPLC–DAD–HESI-HRMS/MS methodology is a new tool that contributes to the understanding of complex samples and their harmful or beneficial metabolism/digestion products. Advantageously, no sample part was lost, and the whole sample was studied without any elaborate sample preparation. Digestion of the oils did not eliminate antibacterial effects or genotoxicity but showed that the metabolism products as well as a genotoxic contaminant may have harmful potential, which requires further investigation and consideration, or even reconsideration of the current risk assessment. Literature about the potential of edible vegetable oils as next-generation antimicrobial agents was confirmed, whereas the observed genotoxic potential remaining after metabolic digestion needs further attention regarding food safety.

## Data availability statement

The original contributions presented in the study are included in the article/Supplementary material, further inquiries can be directed to the corresponding author.

## Author contributions

IM: conceptualization, methodology, experimental analysis, data analysis, and writing—original draft. AG: experimental analysis and data analysis. GM: conceptualization, methodology, supervision, and writing—review and editing. All authors contributed to the article and approved the submitted version.

## Dedication

Dedicated to the lifework of Prof. Dr. Colin Poole, Wayne State University, Detroit, USA.
