# Supplementary material for: Bioactive profiles of edible vegetable oils determined using 10D hyphenated comprehensive high-performance thin-layer chromatography (HPTLC×HPTLC) with on-surface metabolism (nanoGIT) and planar bioassays
Source: Front Nutr. 2023 Sep 22;10:1227546. doi: 10.3389/fnut.2023.1227546 (PMC10556687; doi:10.3389/fnut.2023.1227546)
Supplement: Supplementary file 1 [file Data_Sheet_1.pdf]

Supplementary Material

Bioactive profiles of edible vegetable oils determined using 10D hyphenated comprehensive high-performance thin-layer chromatography (HPTLC×HPTLC) with on-surface metabolism (nanoGIT) and planar bioassays

Isabel Müller[1], Alexander Gulde[1], Gertrud E. Morlock[1.2,#,*]

[1]Institute of Nutritional Science, Chair of Food Science, as well as Interdisciplinary Research Centre for Biosystems, Land Use and Nutrition, Justus Liebig University Giessen, Heinrich-Buff-Ring 26-32, 35392 Giessen, Germany

[2]Center for Sustainable Food Systems, Justus Liebig University Giessen, Senckenbergstr. 3, 35390 Giessen, Germany

[#]Member of the More than One Constituent Substances (MOCS) Initiative, www.vielstoffgemische.de

Dedicated to the lifework of Prof. Dr. Colin Poole, Wayne State University, Detroit, USA

*Corresponding authors: Prof. Dr. Gertrud Morlock, phone: +49-641-9939141; fax +49-641-99-39149, email: gertrud.morlock@uni-giessen.de

Table of contents

**Table S1**	Table S2<br>Supplier information of all investigated oil samples.	4
**Table S2**	Table S3<br>Evaluated masses, substances, and ion species of enzyme interferences via nanoGIT−HPTLC −Vis/FLD−heart cut−RP-HPLC−DAD− HESI-HRMS/MS (Fig. S7).	5
**Table S3**	Table S4<br>Evaluated masses, substances, sum formula and ion species of identified fatty acids after the on-surface digestion of flaxseed oil via nanoGIT−HPTLC×HPTLC−Vis/FLD−heart cut−RP-HPLC−DAD−HESI-HRMS/MS (Fig. S10).	6
**Table S4**	Evaluated masses, substances, sum formula and ion species of identified fatty acids of the standard reference track after subsequent *B. subtilis* assay via HPTLC−Vis/FLD−bioassay−heart cut−RP-HPLC−DAD−HESI-HRMS/MS (Fig. 8).	7
**Table S5**	Evaluated masses, substances, sum formula and ion species of identified fatty acids after the on-surface digestion of coconut oil and subsequent *B. subtilis* assay via nanoGIT−HPTLC×HPTLC−Vis/FLD−bioassay−heart cut−RP-HPLC−DAD−HESI-HRMS/MS (Fig. 9). Retention time shift due to HPLC pump exchange.	8
**Table S1**	Advantages and disadvantages of the developed nanoGIT−HPTLC×HPTLC−Vis/FLD−bioassay−heart cut−RP-HPLC−DAD−HESI-HRMS/MS method in comparison with literature.	9
**Figure S1**	Schemes of the cover plates (HPTLC silica gel 60, layer faced upward) used for neutralization of the HPTLC RP-18 W plates before the application for (**A**) one-dimensional development and (**B**) two-dimensional development.	10
**Figure S2**	Schemes of the cover plates (HPTLC silica gel 60, layer faced upward) used for wetting of the HPTLC RP-18 W plates before the incubation for (**A**) one-dimensional development and (**B**) two-dimensional development.	11
**Figure S3**	Figure S3: Scheme of the focusing process to remove enzyme interferences for the one-dimensional development.	12
**Figure S4**	Figure S4: Scheme and orientation of the two-dimensional development on a 10 cm × 10 cm plate with additional co-development of the standards on a separate plate.	13
**Figure S5**	HPTLC RP-18 chromatogram of triacylglycerols in sunflower (Su), canola (Ca), olive (O), hemp (H), walnut (W), coconut (Co), and flaxseed (F) oil and fatty acid standards of oleic acid (C18:1) and palmitic acid (C16:0), all 10 µg/band each, developed with dichloromethane/acetic acid/acetone 2:4:5 (*V*/*V*/*V*) up to 80 mm, detected at FLD 366 nm after application of the rhodamine 6G reagent (for comparison in Fig. 2, chromatogram with rhodamine 6G reagent and copper sulfate phosphoric acid reagent).	14

**Figure S6**	Figure S6: Influence of the phosphate-citrate buffer pH 12 on HPTLC RP-18 W plates after the derivatization with rhodamine 6G reagent, detected at FLD 366 nm.	15
**Figure S7**	High-resolution mass spectra of the fragmentation of *m/z* 785.5909 and corresponding ion species in the positive ionization mode after nanoGIT−HPTLC −Vis/FLD−heart cut−RP-HPLC−DAD− HESI-HRMS/MS analysis.	16
**Figure S8**	Isotopic pattern of the ion species *m/z* 785.5909 in the positive ionization mode after nanoGIT−HPTLC −Vis/FLD−heart cut−RP-HPLC−DAD−HESI-HRMS/MS analysis revealed a tetramer $[4M+2H]^{+}$ and/or dimer $[2M1+H]^{+}$ at retention time 8.09 min and a dimer $[2M1+H]^{+}$ at retention time 8.44 min.	17
**Figure S9**	High-resolution mass spectra and corresponding ion species in the negative ionization mode after nanoGIT−HPTLC×HPTLC−Vis/FLD−heart cut−RP-HPLC−DAD− HESI-HRMS/MS analysis of all identified fatty acids found in zones **c**, **d** and **e** of digested flaxseed oil on HPTLC RP-18 W plates. Plate focused twice with acetone and cut at 15 mm and developed first with *n*-hexane/diethyl ether/formic acid 90:25:2 (*V*/*V*/*V*) up to 60 mm from cut edge, then turned 90° and developed with acetonitrile/water 4:1 (*V*/*V*) and molecular sieve (3 Å) up to 50 mm.	18

Table S2

Supplier information of all investigated oil samples.

**Vegetable oil**	**Supplier or producer (brand)**	**Best before**	**Production type**
Flaxseed oil	dm Bio, Karlsruhe, Germany	09.2021	Organic
Palm oil	Ölmühle Solling, Boffzen, Germany	10.2022	Organic, red
Hemp oil	Ölmühle Solling, Boffzen, Germany	12.2021	Conventional
Sunflower oil	Huilerie Bio Occitane, Bram, France (Rewe Bio)	06.2022	Organic
Walnut oil	bio Zentrale, Wittibreut, Germany	02.2022	Organic
Coconut oil	Palmin, Elmshorn, Germany	Expired	Conventional
Olive oil	El. Renieris & Co., Kissamos/Crete, Greece (Lidl, Eridanous)	01.2022	Conventional, extra native
Canola oil	Mazola, Elmshorn, Germany	Expired	Conventional
Soybean oil	Heuschen & Schrouff, Landgraaf, Netherlands	05.2022	Conventional

Table S3

Evaluated masses, substances, and ion species of enzyme interferences via nanoGIT−HPTLC −Vis/FLD−heart cut−RP-HPLC−DAD− HESI-HRMS/MS (Fig. S7).

**RT [min]**	**Substance**	***m/z***	**Mass error [Δ ppm]**	**Ion species**
8.09 (ESI^+)<br>8.11 (ESI^-)	UDCA, HDCA, CDCA, DCA	391.2858	−2.57	$[M1-H]^-$
		437.2913	−2.28	$[M1+HCOO]^-$
		451.3070	−2.36	$[M1+CH_3\text{-}COO]^-$
		783.5791	−2.02	$[2M1-H]^-$
		785.5909	2.18	$[2M1+H]^+$<br>$[4M1+2H]^+$
	Cholenic acid	357.2780	2.20	$[M2-H_2O+H]^+$
		375.2885	2.45	$[M2+H]^+$
		392.3149	2.57	$[M2+NH_4]^+$
8.45	UDCA, HDCA, CDCA, DCA	391.2860	−3.05	$[M1-H]^-$
		437.2916	−1.61	$[M1+HCOO]^-$
		451.3072	−1.48	$[M1+CH_3\text{-}COO]^-$
		783.5794	-1.73	$[2M1-H]^-$
		393.2988	2.91	$[M1+H]^+$
		410.3266	−0.35	$[M1+NH_4]^+$
		785.5927	−0.15	$[2M1+H]^+$
	UDCA, HDCA, CDCA, DCA, Cholenic acid	357.2790	−0.40	$[M1-2H_2O+H]^+$
				$[M2-H_2O+H]^+$
		375.2894	−0.05	$[M1-H_2O+H]^+$
				$[M2+H]^+$

M1 = UDCA, HDCA, CDCA, DCA; **M2** = Cholenic acid; **UDCA**: Ursodeoxycholic acid; **HDCA**: Hyodeoxycholic acid; **(C)DCA**: (Cheno)deoxycholic acid

Table S4

Evaluated masses, substances, sum formula and ion species of identified fatty acids after the on-surface digestion of flaxseed oil via nanoGIT−HPTLC×HPTLC−Vis/FLD−heart cut−RP-HPLC−DAD−HESI-HRMS/MS (Fig. S10).

**Zone**	**RT [min]**	**Substance**	**Sum formula**	***m/z***	**Mass error [Δ ppm]**	**Ion species**
c	6.71	Oxidized C9:0	C9 H15 O3	171.1028	−0.77	$[M3-H]^-$
	7.49	Oxidized C12:1	C12 H19 O3	211.1340	−0.15	$[M4-H]^-$
d	6.71	Oxidized C9:0	C9 H15 O3	171.1028	−0.77	$[M3-H]^-$
	7.49	Oxidized C12:1	C12 H19 O3	211.1340	−0.15	$[M4-H]^-$
	9.03	C14:0	C14 H27 O2	227.2018	−0.64	$[M5-H]^-$
		C18:3	C18 H29 O2	277.2175	−0.71	$[M6-H]^-$
	9.24	C18:2	C18 H31 O2	279.2334	−1.60	$[M7-H]^-$
	9.38	C16:0	C16 H31 O2	255.2334	−1.75	$[M8-H]^-$
	9.49	C18:1	C18 H33 O2	281.2489	−1.05	$[M9-H]^-$
e	8.29	C10:0	C10 H19 O2	171.1391	−0.27	$[M10-H]^-$
	8.51	C11:0	C11 H21 O2	185.1546	0.56	$[M11-H]^-$
	8.68	C12:0	C12 H23 O2	199.1707	−1.74	$[M12-H]^-$

M3 = oxidized C9:0, **M4** = oxidized C12:1, **M5** = C14:0, **M6** = C18:3, **M7** = C18:2, **M8** = C16:0, **M9** = C18:1, **M10** = C10:0, **M11** = C11:0, **M12** = C12:0

Table S5

Evaluated masses, substances, sum formula and ion species of identified fatty acids of the standard reference track after subsequent *B. subtilis* assay via HPTLC−Vis/FLD−bioassay−heart cut−RP-HPLC−DAD−HESI-HRMS/MS (Fig. S11).

Zone	RT [min]	Substance	Sum formula	*m/z*	Mass error [Δ ppm]	Ion species
c	9.01	C14:0	C14 H27 O2	227.2017	−0.07	$[M5-H]^-$
	9.22	C18:2	C18 H31 O2	279.2328	0.55	$[M7-H]^-$
e	8.72	C12:0	C12 H23 O2	199.1702	0.77	$[M12-H]^-$
	9.07	C18:3	C18 H29 O2	277.2174	−0.35	$[M6-H]^-$
f	7.81	C8:0	C8 H15 O2	143.1077	0.38	$[M13-H]^-$
	8.33	C10:0	C10 H19 O2	171.1392	−0.85	$[M10-H]^-$

M5 = C14:0, **M6** = C18:3, **M7** = C18:2, **M10** = C10:0, **M12** = C12:0, **M13** = C8:0

Table S6

Evaluated masses, substances, sum formula and ion species of identified fatty acids after the on-surface digestion of coconut oil and subsequent *B. subtilis* assay via nanoGIT−HPTLC×HPTLC−Vis/FLD−bioassay−heart cut−RP-HPLC−DAD−HESI-HRMS/MS (Fig. S12). Retention time shift due to HPLC pump exchange.

**Zone**	**RT [min]**	**Substance**	**Sum formula**	***m*/*z***	**Mass error [Δ ppm]**	**Ion species**
c	9.86	C14:0	C14 H27 O2	227.2019	−0.95	$[M5-H]^-$
e	9.69	C12:0	C12 H23 O2	199.1703	0.42	$[M12-H]^-$
f	9.15	C10:0	C10 H19 O2	171.1389	0.96	$[M10-H]^-$

M5 = C14:0, **M10** = C10:0, **M12** = C12:0

Table S7

Advantages and disadvantages of the developed nanoGIT−HPTLC×HPTLC−Vis/FLD−bioassay−heart cut−RP-HPLC−DAD−HESI-HRMS/MS method in comparison with literature

<table>
<tr><th>Method highlights</th><th>Compared method</th><th>Advantages</th><th>Disadvantages</th></tr>
<tr><td>All-in-one system (metabolization + analysis)</td><td rowspan="2"><u>TAG analysis</u>
GC-FID (29)
<u>Fatty acid analysis</u>
- HPLC-ELSD (23)
- GC-FID (23, 29)
<u>Sum parameter value</u>
- Spectrophotometric assay kit (26)
- pH-stat titration (29)
- GC-FID (29)</td><td>- No sample loss
- No sample preparation after metabolization
- Miniaturized approach (less amounts, more sustainable, faster metabolization)</td><td></td></tr>
<tr><td>Analysis of detailed acylglycerol composition with subsequent detailed fatty acid composition</td><td>- Separation of TAGs, DAGs, MAGs, and FAs in one run
- No sample loss
- No fatty acid methyl esters needed
- No pre-chromatographic derivatization step
- Evaluation of sum parameter values possible if calibration on plate
- Subsequent detailed FA analysis possible</td><td>- No detailed TAG composition</td></tr>
<tr><td>Application of bioassays and hyphenation with HRMS/MS</td><td>Disk diffusion (58)
Broth microdilution (59)</td><td>- No sample loss
- Detailed analysis and identification of the individual bioactive substance in the food matrix
- Identification of signal-responsible substances after the bioassay</td><td></td></tr>
</table>

GC-FID = gas chromatography with flame ionization detector, HPLC-ELSD = high-performance liquid chromatography with evaporative light scattering detector, TAG = triacylglycerol, DAG = diacylglycerol, MAG = monoacylglycerol, FA = fatty acid

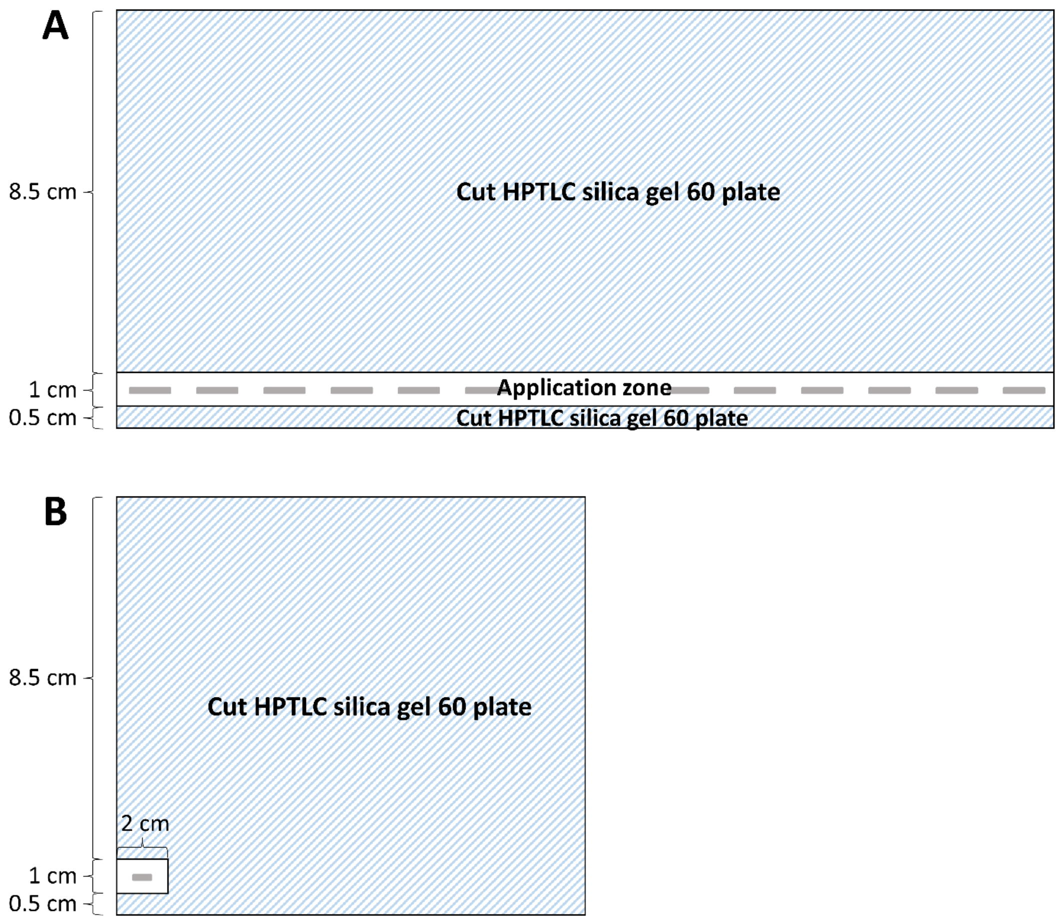

Figure S1: Schemes of the cover plates (HPTLC silica gel 60, layer faced upward) used for neutralization of the HPTLC RP-18 W plates before the application for (**A**) one-dimensional development and (**B**) two-dimensional development.

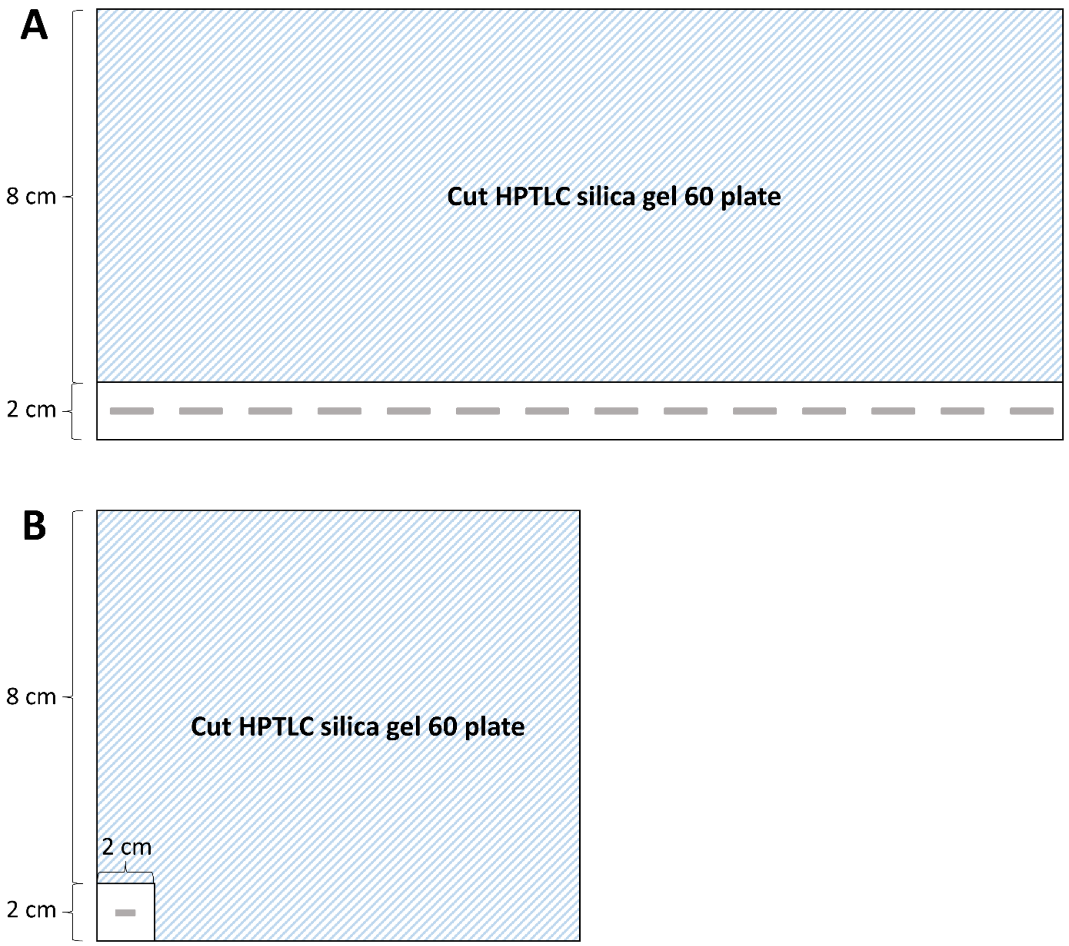

Figure S2: Schemes of the cover plates (HPTLC silica gel 60, layer faced upward) used for wetting of the HPTLC RP-18 W plates before the incubation for (**A**) one-dimensional development and (**B**) two-dimensional development.

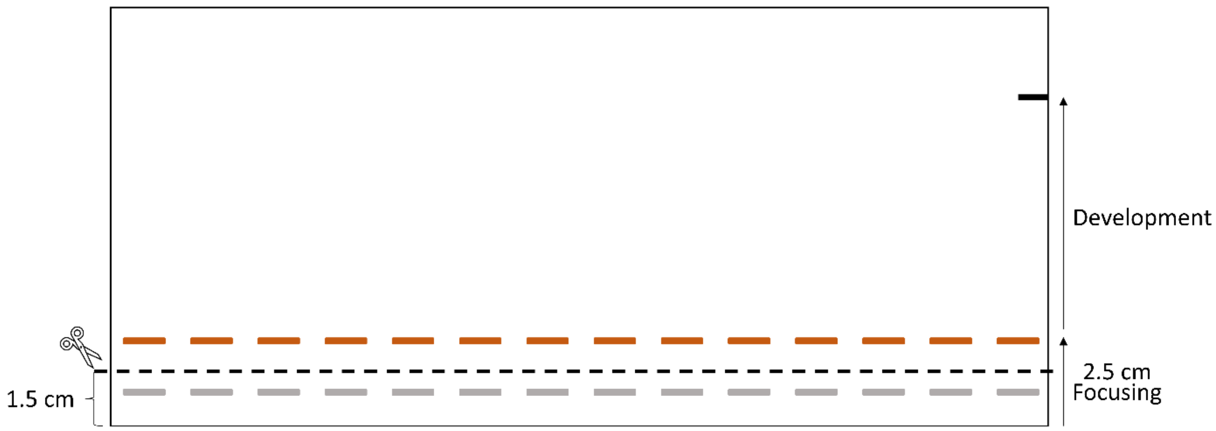

Figure S3: Scheme of the focusing process to remove enzyme interferences for the one-dimensional development.

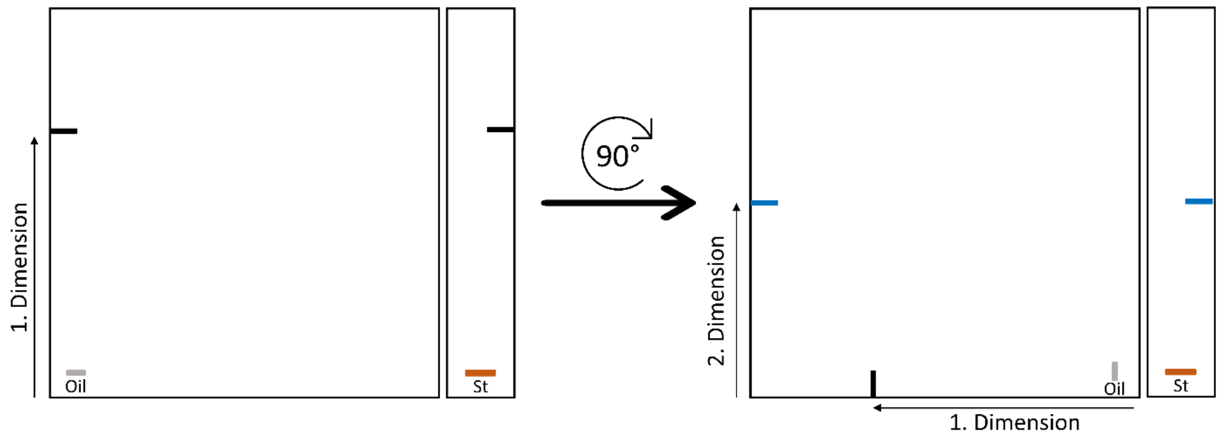

Figure S4: Scheme and orientation of the two-dimensional development on a 10 cm × 10 cm plate with additional co-development of the standards on a separate plate.

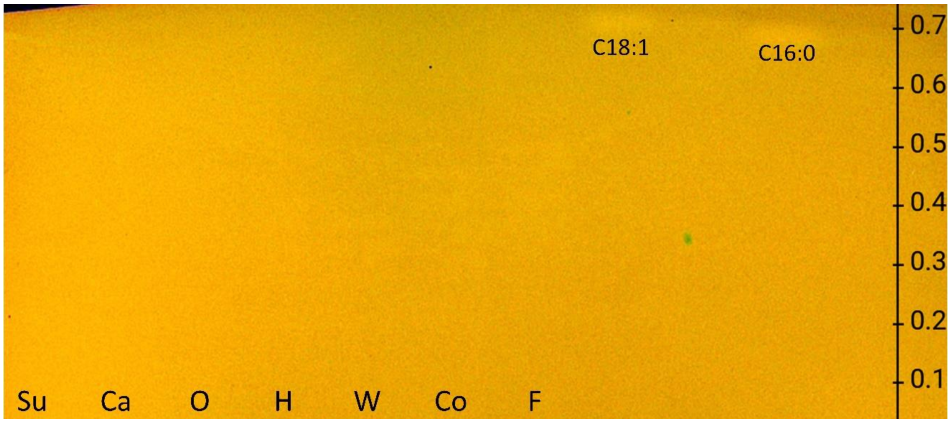

Figure S5: HPTLC RP-18 chromatogram of triacylglycerols in sunflower (Su), canola (Ca), olive (O), hemp (H), walnut (W), coconut (Co), and flaxseed (F) oil and fatty acid standards of oleic acid (C18:1) and palmitic acid (C16:0), all 10 µg/band each, developed with dichloromethane/acetic acid/acetone 2:4:5 (*V*/*V*/*V*) up to 80 mm, detected at FLD 366 nm after application of the rhodamine 6G reagent (for comparison in Fig. 2, chromatogram with rhodamine 6G reagent and copper sulfate phosphoric acid reagent).

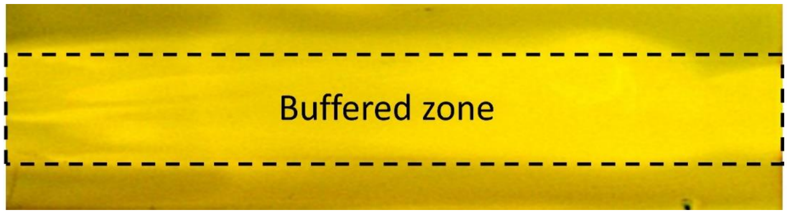

Figure S6: Influence of the phosphate-citrate buffer pH 12 on HPTLC RP-18 W plates after the derivatization with rhodamine 6G reagent, detected at FLD 366 nm.

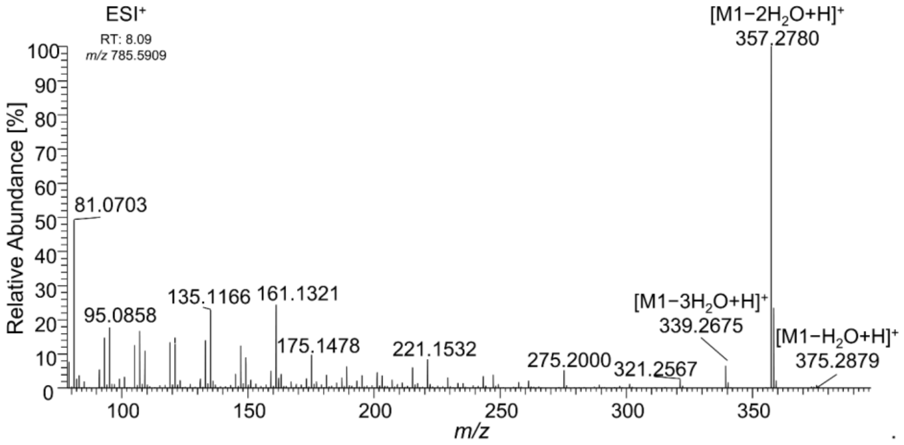

Figure S7: High-resolution mass spectra of the fragmentation of *m/z* 785.5909 and corresponding ion species in the positive ionization mode after nanoGIT−HPTLC −Vis/FLD−heart cut−RP-HPLC−DAD− HESI-HRMS/MS analysis. **M1** = Ursodeoxycholic acid, hyodeoxycholic acid, (cheno)deoxycholic acid

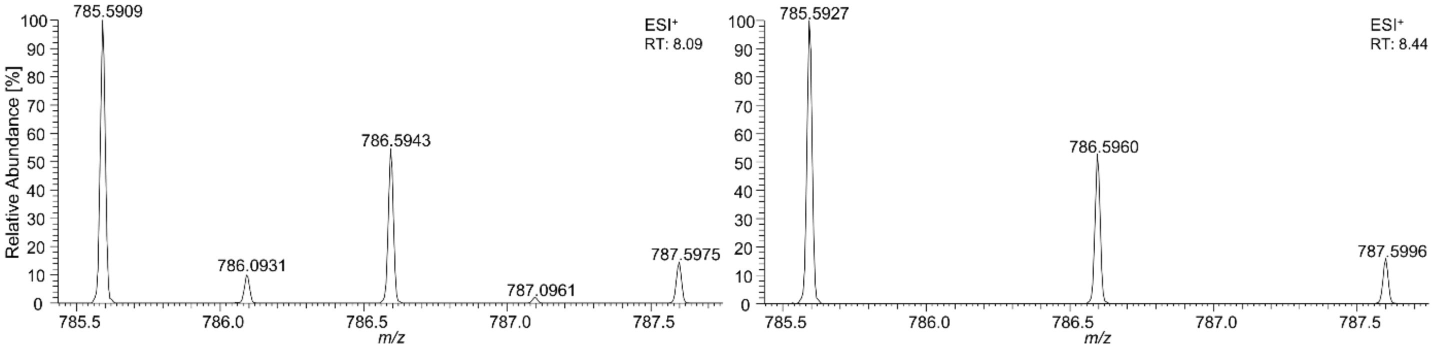

Figure S8: Isotopic pattern of the ion species *m/z* 785.5909 in the positive ionization mode after nanoGIT−HPTLC −Vis/FLD−heart cut−RP-HPLC−DAD− HESI-HRMS/MS analysis revealed a tetramer $[4M+2H]^+$ and/or dimer $[2M1+H]^+$ at retention time 8.09 min and a dimer $[2M1+H]^+$ at retention time 8.44 min. **M1** = Ursodeoxycholic acid, hyodeoxycholic acid, (cheno)deoxycholic acid

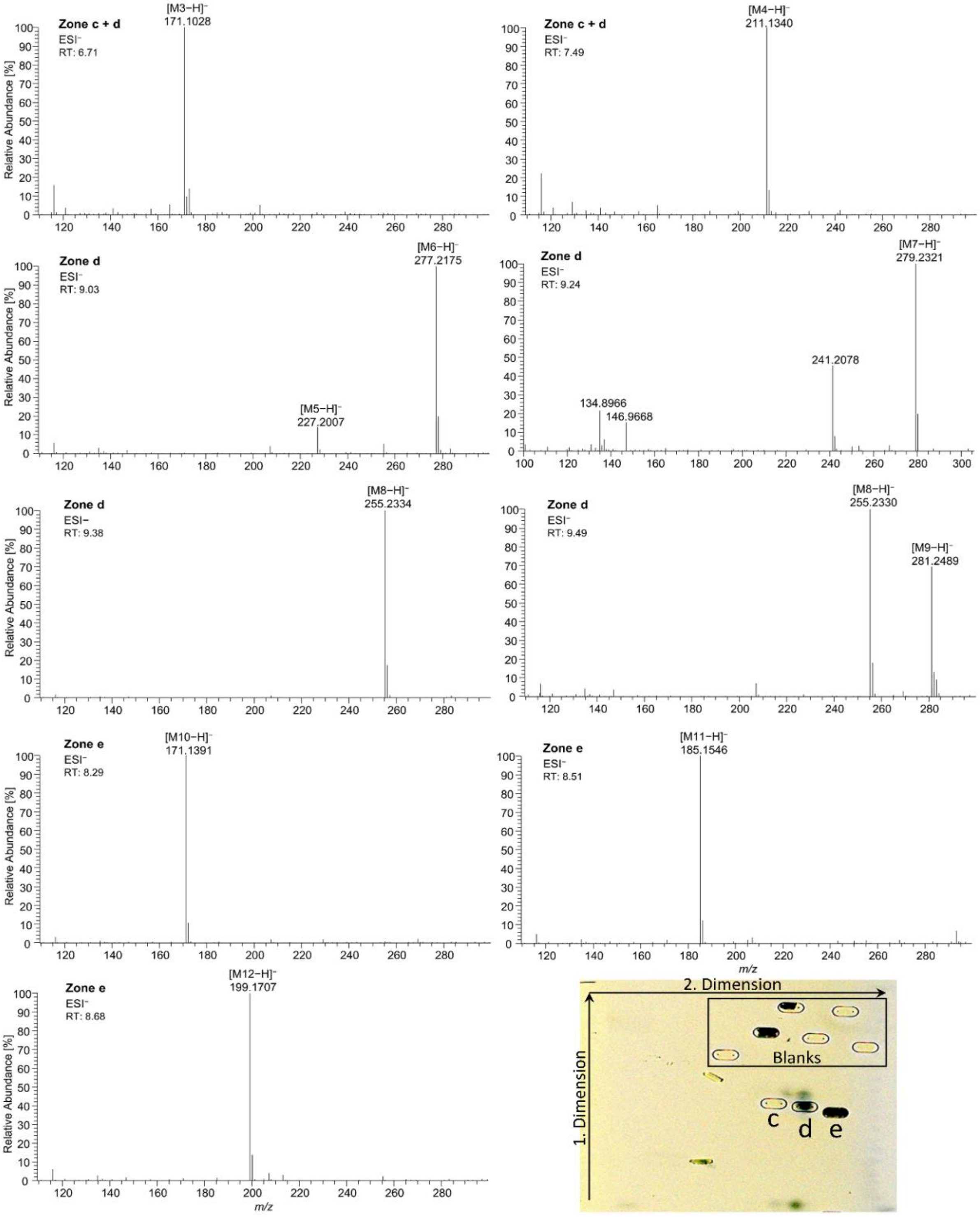

Figure S9: High-resolution mass spectra and corresponding ion species in the negative ionization mode after nanoGIT−HPTLC×HPTLC−Vis/FLD−heart cut−RP-HPLC−DAD− HESI-HRMS/MS analysis of all identified fatty acids found in zones **c**, **d** and **e** of digested flaxseed oil on HPTLC RP-18 W plates. Plate focused twice with acetone and cut at 15 mm and developed first with *n*-hexane/diethyl ether/formic acid 90:25:2 (*V*/*V*/*V*) up to 60 mm from cut edge, then turned 90° and developed with acetonitrile/water 4:1 (*V*/*V*) and molecular sieve (3 Å) up to 50 mm; after the transfer of the interesting zones to the HRMS, the stamped plate was derivatized using the copper sulfate phosphoric acid reagent to check whether the elution head was properly positioned on the zones of interest. **M3** = oxidized C9:0, **M4** = oxidized C12:1, **M5** = C14:0, **M6** = C18:3, **M7** = C18:2, **M8** = C16:0, **M9** = C18:1, **M10** = C10:0, **M11** = C11:0, **M12** = C12:0
